# Reflecting on LLM Support in Reflexive Thematic Analysis: An Exploratory Study

**DOI:** 10.1177/10497323251365211

**Published:** 2025-09-08

**Authors:** Magnhild Vikan, Ramtin Aryan, Mari Serine Kannelønning, Michael Alexander Riegler, Stein Ove Danielsen

**Affiliations:** 1 Department of Nursing and Health Promotion, Faculty of Health Sciences, 60499 Oslo Metropolitan University, Oslo, Norway; 2 Department of Information and Communication Technology, Division Organization and Infrastructure, 60499 Oslo Metropolitan University, Oslo, Norway; 3 The University Library, 60499 Oslo Metropolitan University, Oslo, Norway; 4 Simula Cyber Security, 394155 Simula Research Laboratory, Oslo, Norway

**Keywords:** qualitative analysis, reflexive thematic analysis, reflexivity, exploratory study, health science, artificial intelligence, large language model

## Abstract

The launch of ChatGPT in November 2022 accelerated discussions and research into whether base large language models (LLMs) could increase the efficiency of qualitative analysis phases or even replace qualitative researchers. Reflexive thematic analysis (RTA) is a commonly used method for qualitative text analysis that emphasizes the researcher’s subjectivity and reflexivity to enable a situated, in-depth understanding of knowledge generation. Researchers appear optimistic about the potential of LLMs in qualitative research; however, questions remain about whether base models can meaningfully contribute to the interpretation and abstraction of a dataset. The primary objective of this study was to explore how LLMs may support an RTA of an interview text from health science research. Secondary objectives included identifying recommended prompt strategies for similar studies, highlighting potential weaknesses or challenges, and fostering engagement among qualitative researchers regarding these threats and possibilities. We provided the interview file to an offline LLM and conducted a series of tests aligned with the phases of RTA. Insights from each test guided refinements to the next and contributed to the development of a recommended prompt strategy. At this stage, base LLMs provide limited support and do not increase the efficiency of RTA. At best, LLMs may identify gaps in the researchers’ perspectives. Realizing the potential of LLMs to inspire broader discussion and deeper reflections requires a well-defined strategy and the avoidance of misleading prompts, self-referential responses, misguiding translations, and errors. Conclusively, high-quality RTA requires a human, comprehensive familiarization phase, and methodological competence to preserve epistemological integrity.

## Background

The body of literature examining how the emergence of large language models (LLMs) influences healthcare science and research methods has grown substantially (Christou, 2023; Owoahene Acheampong & Nyaaba, 2024; Sallam, 2023; van Manen, 2023). LLMs became common knowledge globally when OpenAI launched the first version of ChatGPT in November 2022 (OpenAI, 2025). The models are generative AI based on deep learning, designed to simulate the human brain’s neural network by creating sets of artificial neurons (OpenAI, 2025; Raiaan et al., 2024; Ray, 2023). They are pretrained using a self-supervised learning approach and introduced to a massive amount of text to develop neural networks of billions of parameters (Christou, 2023; Raiaan et al., 2024). The training aims to teach LLMs to understand and generate human-like language, complex content patterns, text synthesis, summaries, and sentiment analyses (Christou, 2024; Raiaan et al., 2024). LLMs create output based on the statistical linguistic predictability of the likelihood of specific words appearing in proximity to one another (OpenAI, 2025; Raiaan et al., 2024).

The use of LLMs in research writing may improve papers by increasing readability and linguistic variations, thus rendering them more informative (Hadan et al., 2024; Sallam, 2023). A recent review of LLM as a tool for generating systematic reviews reported promising results regarding the efficiency and guidance of scientific review processes (Scherbakov et al., 2024). Additionally, LLMs may assist in evaluating multiple-choice answers, though the models have been demonstrated to struggle with processing long texts and providing critical responses (Zhou et al., 2024). Morgan (2023) questioned whether LLMs might challenge the existing dominance of coding as a paradigm in qualitative analysis. A recent review suggested that LLMs could manage large datasets, facilitate brainstorming, and guide research designs; however, the review pointed to a need for research and a framework for integrating LLMs into qualitative studies, particularly in addressing ethical challenges (Owoahene Acheampong & Nyaaba, 2024).

It is important to explore the effectiveness and capacity of LLMs in comparison to the time-consuming nature of traditional qualitative processes (Dai et al., 2023; Hitch, 2023; Morgan, 2023; Perkins & Roe, 2024). Previous studies have reported that LLMs can be useful and efficient regarding initial coding (Dai et al., 2023; Morgan, 2023). Engstrom et al. (2022) compared a semi-automated content analysis with a manual one. The semi-automated analysis took 21 hours, compared to 73 hours for the manual analysis, and achieved 74% concordance with the manual results (Engstrom et al., 2022). Perkins and Roe (2024) compared a ChatGPT-based analysis with a human-led thematic analysis, highlighting LLMs’ enormous capacity to identify themes in large datasets. The same study noted the risk of errors in responses, making them challenging to manage (Perkins & Roe, 2024). Wachinger et al. (2024) conducted one interview to test whether an LLM could perform a qualitative analysis. They reported that ChatGPT or similar models might support qualitative analysis, particularly in identifying and linking themes to theories, but also noted that LLMs sometimes link themes to irrelevant theories (Wachinger et al., 2024).

LLMs appear more likely to produce descriptive summaries than to generate interpreted and abstracted themes of meaning (Morgan, 2023; Perkins & Roe, 2024; Wachinger et al., 2024). LLM-generated texts may lack details and the reflective nuance characteristic of human analysis (Hadan et al., 2024). Maintaining coherence across extensive datasets and long LLM chats might be challenging for the models (Lindebaum & Fleming, 2024; Raiaan et al., 2024). Engstrom et al. (2022) found that automated tools failed to identify concepts requiring emotional and contextual understanding. This finding is supported by other studies reporting that LLMs could not reflect and interpret underlying cultural, situated, and contextual meanings (Christou, 2024; Hitch, 2023; Morgan, 2023; Perkins & Roe, 2024); these skills are core values of reflexivity in reflexive thematic analysis (RTA) (Braun & Clarke, 2006, 2022).

RTA is the most commonly used approach to thematic analysis in qualitative research (Braun & Clarke, 2006). The method falls within the Big Qualitative (BigQ) tradition, which refers to qualitative research grounded in the interpretative paradigm and a qualitative set of values (Braun & Clarke, 2013, 2022). BigQ highlights the researcher’s subjectivity, sensibility, and contextual understanding (Braun & Clarke, 2013, 2022). Subjectivity involves integrating the researcher’s perspectives, politics, and passion into the analysis, while sensitivity entails interest in process and meaning, a critical approach to knowledge, and the ability to challenge one’s pre-assumptions (Braun & Clarke, 2013). The researcher’s reflexivity is emphasized to enable a broad, in-depth understanding and the active generation of themes (Braun & Clarke, 2013, 2022). RTA is an iterative sense-making process described in six phases, detailed in Table 1 (Braun & Clarke, 2006).

**Table 1.** Reflexive Thematic Analysis.

Phases in reflexive thematic analysis
1. Familiarization involves becoming deeply familiar with the data through reading, listening, and writing brief analytical insights.
2. Coding involves identifying meaningful data segments. Code labels should describe a single semantic or latent meaning or idea.
3. Generation of initial themes involves searching for patterns of meaning across the dataset and compiling similar codes into themes.
4. Development and review of themes involves assessing how the initial themes display the most important patterns of meaning and tell a compelling story.
5. Refinement, definition, and naming of themes involves demarcating themes built upon a strong core concept or essence and giving them punchy names.
6. Writing-up involves creating a coherent story to meet the study’s objectives.

How to use LLMs to identify underlying patterns of meaning and text interpretation remains an open question (Morgan, 2023). Therefore, we need to explore the possibilities of using LLMs in the qualitative analysis of large datasets designed for real-life research purposes rather than for AI investigations alone. The primary objective of this study was to establish a baseline by exploring and reflecting on how base LLMs, without reasoning or deep research capabilities, may support researchers in the analytical process of RTA by reanalyzing an interview text from the health science field. The secondary objectives were to reflect on and recommend a prompt strategy that could enhance LLMs’ analytical support, identify potential challenges and weaknesses, and foster engagement and reflections among qualitative researchers regarding these threats and possibilities. Our research question was: “How can a base LLM support human researchers in conducting an RTA of an interview text?”

## Methods

### Study Design

This study employed an exploratory design. The first and last authors adopted an inductive approach to explore how LLM could support the RTA process. We derived the dataset from our previously published qualitative study in the health science field. The primary study aimed to explore surgical team members’ perspectives on patient safety culture in the surgical context (Vikan et al., 2024). We analyzed the data in the primary study by using Braun and Clarke’s method for RTA, following a BigQ abductive approach (2006, 2022). The dataset consisted of 223 pages of verbatim interview transcripts, totaling 158,865 words (Vikan et al., 2024).

### The Dataset

We used the interview data of a single participant from the primary dataset to conduct this study. This interview was a 10-page text file comprising 9262 words, originally written in Norwegian. The participant was an operating room nurse, and the content of the file displayed the themes and subthemes generated during the primary analysis (Vikan et al., 2024). We cleaned the interview text by removing the moderator’s questions and the transcription symbols indicating pauses and silences (Braun & Clarke, 2013).

### The Research Team

We strategically assembled a multidisciplinary research team to achieve the study’s objectives. The first and last authors were operating room nurses with in-depth knowledge of the surgical context, patient safety culture, the primary dataset, and RTA. The first author was a PhD candidate, and the last was the supervisor and an associated professor. The second author was a computer researcher who proposed suitable methods and platforms for analyzing the interview file, reviewed the results, and provided feedback on the prompts. The third author was a social scientist and senior research librarian with expertise in AI use and AI literacy in research practices. The fourth was a professor and senior researcher in AI. The first and last authors held introductory, reflexive meetings with the other authors to establish a shared understanding before initiating the testing. As a shared stance, the authors considered LLMs to be technical tools and were critical of any humanization of these models. For this reason, we used the term “errors” instead of the humanized term “hallucination” throughout this paper. At the same time, we remained curious and open-minded about the potential of LLMs to support interpretative qualitative analysis of underlying meanings.

### Technical Factors

The second author supported the technical considerations and resolutions. Due to the ethical aspects of privacy and data protection in the interview text from health science research, we used an offline LLM, which required a powerful personal computer (GPU: NVIDIA GeForce RTX 4070 Ti Super, CPU: AMD Ryzen 7 8-Core, RAM: 32 GB). We created a virtual environment using the Ollama platform (Ollama, 2025) and downloaded LLMs from Hugging Face. After conducting Test 1 across multiple models, we found that the LLM Mistral-7B, with seven billion parameters and default settings, provided the most meaningful responses (see details in Supplemental Materials 1) (Hugging Face, 2024). We selected this foundational offline model to prioritize data security and to assess the core text-generation capabilities of the underlying technology. The offline LLM was pretrained on a comprehensive dataset and generated responses locally without internet access. A key technical consideration was the model’s context length, meaning the working memory and how much of the input text or conversation it could retain and use to inform its responses. This setup allowed us to leverage the retrieval-augmented generation (RAG) technique to incorporate the interview text file and test it alongside additional files, such as the RTA and theories of patient safety culture and quality in healthcare, to support a more comprehensive analysis.

### Testing and Data Collection

We initially planned the main steps of the systematic exploration and prompt strategies based on previous literature and developed an approach based on each phase of the RTA method, as presented in Table 1 (Bsharat et al., 2023; Christou, 2023; Hitch, 2023; Khurana et al., 2024; Morgan, 2023; Wachinger et al., 2024; Zhang et al., 2023). This approach was also informed by less structured initial tests, which allowed us to familiarize ourselves with the LLM tool. In one of these initial tests, we attempted a complete analysis using a single prompt that explained the RTA phases. Another initial test involved a strategy created by ChatGPT. These initial tests produced incoherent responses and did not contribute to the analysis; thus, we adopted a stepwise testing approach based on RTA phases.

**Figure 1.**
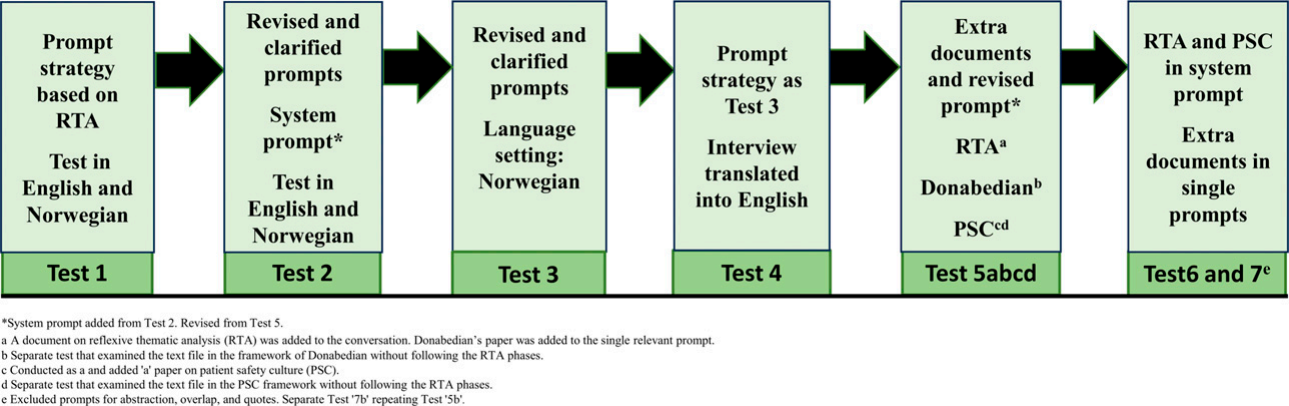
Prompting strategy in Tests 1–7.

We developed the inductive approach and testing strategy by evaluating and reflecting on the LLM’s output, identifying potential errors or challenges, and comparing the responses to the primary study’s results and our in-depth understanding of the raw data (Vikan et al., 2024). We wrote reflexive memos throughout the process to document reflections and deepen our understanding of the prompts, responses, errors, and potential threats to trustworthiness. Although the process is presented linearly, each test represented a circular, iterative, and interpretative process aligned with BigQ values. The process also included smaller creative exploratory tests that did not significantly contribute to the final results. The first author conducted the testing and wrote the memos to encourage critical reflections and discussion with the last author, while also receiving technical support from the computer researcher. The recommended strategy was primarily developed through seven tests, presented in Figure 1, each followed by a reflexive meeting to evaluate the responses and plan the next test. The testing period spanned from November 11, 2024, to January 6, 2025.

### Prompting Strategy in Stepwise Testing

We attached the interview text file to all conversations in the testing process, prompting the LLM to generate responses based on its content.

#### Test 1

To begin inductively, Test 1 was conducted without a system prompt. In the conversation prompt, we assigned the LLM the role of a scientist and researcher performing qualitative analysis and instructed it to write to an audience of scientists conducting qualitative research. We attached the interview file in Norwegian and prompted the model to generate answers in the same language. Additionally, we informed the model that the text file contained an interview with a surgical team member discussing perspectives on patient safety in a surgical context. Due to the exploratory and inductive design, and to avoid introducing leading or confusing concepts, the model was not exposed to the concept of patient safety culture or to RTA as a method until Test 5. To test the model’s support for familiarization, we requested a 150-word summary of the interview text. We then guided the LLM through the RTA phases, including prompts for abstraction, theme interactions and overlap, illustrative quotes, linkages to relevant and irrelevant theories, and implications for clinical performance and research. We repeated Test 1 and prompted for English responses to explore this LLM’s ability to translate the interview text (see Supplemental Materials 1 and 2).

#### Test 2

From Test 2 onward, we formulated a system prompt for each conversation. This prompt described our roles, the interview file, the topic, and the context as in the first conversation prompt in Test 1, and we prompted Norwegian responses. Based on the Test 1 responses, we refined the prompts linguistically, clarifying and specifying them where needed. Some prompts were refined in detail, while others were simplified. To test whether the model identified the content’s underlying meaning, we divided the coding prompt into two parts: one for generating codes based on semantic content and another for latent content. We also requested more in-depth descriptions of the themes and excluded the prompt that asked the model to link the interview text to an irrelevant theory. Test 2 was also repeated in English to explore the trustworthiness of the translation and to identify potential translation issues (see Supplemental Materials 3 and 4).

#### Test 3

For Test 3, we used the same system prompt and further clarified the conversation prompts. Additionally, we specified Norwegian as the preferred dictionary and language setting. We requested a summary of 300 words instead of 150 to explore whether the response covered more of the content from the interview text and supported the familiarization further. Based on the LLM’s tendency to mix latent and semantic codes in Test 2, we merged these prompts into one. We also combined the prompts for generating and describing subthemes and themes to test whether this improved the coherence of the themes and the descriptions (see Supplemental Materials 5).

#### Test 4

In Test 4, we used the same system and conversation prompts as in Test 3. Based on the limited substance and analytical support in Tests 1–3, we translated the interview text into English, the language in which our offline LLM was primarily trained. We translated the document using another LLM with stronger Norwegian language capabilities, and all data privacy and protection measures were followed in accordance with the regulations of the authors’ organization (Sikt, 2024). A fluent English-speaking human translator reviewed and quality checked the translation. We changed the language setting to English Great Britain (see Supplemental Materials 6). Based on the more credible responses obtained, we used this translated document and language settings in Tests 5–7.

#### Test 5a, 5b, 5c, and 5d

As recommended in previous literature, we included phrases such as “Support phase 1 in the analysis,” “Support phase 2 in the analysis,” and so on in the conversation prompts for Test 5a (Bsharat et al., 2023). We updated the system prompt to specify that the qualitative analysis method was RTA, attached a document describing the method (Braun & Clarke, 2006) to the conversation, and instructed the LLM to base its responses on this document. Donabedian’s theory of quality in healthcare served as the theoretical framework for the primary study. Accordingly, we prompted the model to relate the data to this framework and attached a document describing the theory to that specific prompt (see Supplemental Materials 7) (Donabedian, 1966). Test 5b, a shorter test, examined how the LLM related the interview file to Donabedian’s theory. We attached the theory document and used the same system prompt as in Tests 2–4. A second conversation prompt was added to explore the linkage in more detail (see Supplemental Materials 8). In Test 5c, we expanded on Test 5a by including the concept of patient safety *culture* in the system prompt and attaching a document outlining the dimensions of patient safety culture (Churruca et al., 2021). We then prompted the LLM to examine the interview text in relation to Donabedian’s theory, without attaching an additional document for this theory to this test (see Supplemental Materials 9). Test 5d, another short test, examined the text file within the framework of patient safety culture dimensions, without guiding the LLM through the RTA phases (see Supplemental Materials 10). We attached a document describing the framework and used the system prompt from Tests 2–4 (Churruca et al., 2021).

#### Test 6

We retained the specification of RTA as the analytical method in the system prompt but chose not to attach documents on the method, as the LLM became overly focused on analytical procedures. We included the concept of patient safety *culture* to the system prompt and attached a document outlining the dimensions of patient safety culture to a single prompt in the conversation (Churruca et al., 2021). Similarly, we attached a document on Donabedian’s theory to this single prompt, rather than to the entire conversation, to avoid confusing the model’s text generation. To increase coherence throughout the conversation, we repeated the themes and subthemes generated by the LLM in subsequent prompts (see Supplemental Materials 11).

#### Test 7a and 7b

We conducted Test 7a in the same way as Test 6. However, we excluded the prompts about abstractions, quotes, and interrelation or overlap between themes, based on the limited contribution in previous tests. As a separate conversation, Test 7b revisited the relationship between the interview text and Donabedian’s theory. The rationale for this test was the varied results observed in earlier tests. We attached a document outlining the theory to support the analysis (see Supplemental Materials 12) (Donabedian, 1966).

### Reflexive Processes and Outcomes of Testing

We summarized the testing process, responses, and reflections, laying the foundation for a recommended prompt strategy for LLM-supported RTA. In doing so, we also identified several challenges and risks of error associated with using LLMs to support analytical processes. Additionally, the results from the primary study, along with the authors’ in-depth understanding of the raw data and supplementary documents, informed discussions about the quality and trustworthiness of the LLM-generated responses (Braun & Clarke, 2006; Churruca et al., 2021; Donabedian, 1966; Vikan et al., 2024). Braun and Clarke emphasize that there is no single “right” result in RTA; the results are generated through an iterative, reflexive process grounded in BigQ values and may lead to different themes across research groups (Braun & Clarke, 2022).

### Ethical Considerations

The Norwegian Agency for Shared Services in Education and Research (Sikt) approved the secondary analysis of the interview data. This approval ensured the protection of participants’ privacy and data, in compliance with the ethical responsibilities outlined in the Helsinki Declaration (World Medical Association, 2024). The first author provided all participants from the primary study with written digital information about the LLM-based analysis of the dataset, including details on privacy, data protection, and voluntariness (see Supplemental Materials 13). Participants returned encrypted written consent, which we stored on a server designated for sensitive data. To ensure data privacy and protect the unidentified dataset, we used an offline platform for all analyses.

## Results and Reflections

The exploratory tests resulted in prompts, responses, and reflecting memos in a Word document of 50,009 words (see Supplemental Materials 1–12). The results summarize how LLMs might support human researchers in RTA. The LLM provided more credible responses after translating the interview text into English, the language in which it was trained. Responses in Norwegian or based on the Norwegian transcript were of poor quality regarding content coverage, the occurrence of errors, and linguistic formulations. For example, in a Norwegian summary, the LLM’s summary generates, “A patient has a splint placed in the femur, two centimeters from the main artery.” This response contains an error and does not accurately convey the content’s meaning. Comparably, in an English summary, the LLM generates, “The team emphasizes the importance of communication, as it contributes significantly to patient positioning decisions.” The detail about the splint highlighted in the Norwegian summary is a description of the collaboration, as shown in the English summary. Thus, LLMs appear to be sensitive to translation, and meaning might be changed or lost.

**Figure 2.**
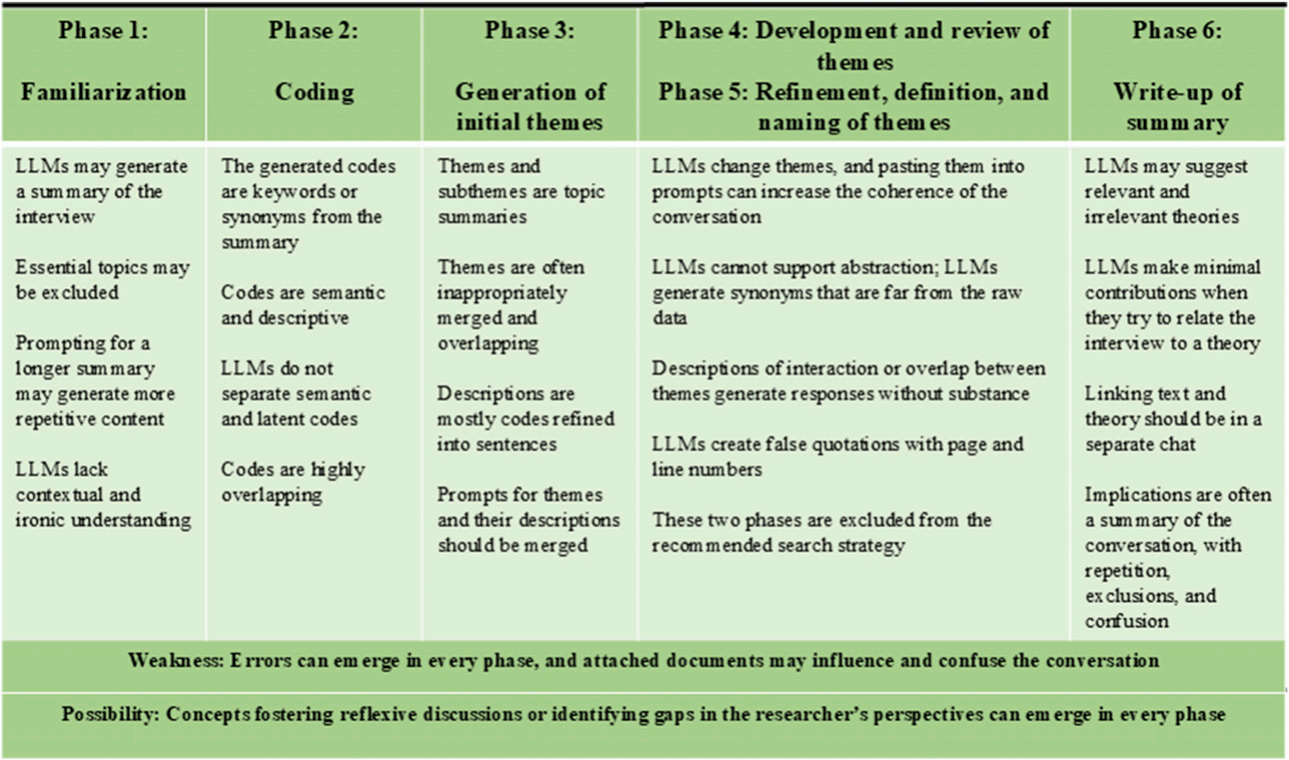
Main results.

The prompt strategies developed and became more specific, concrete, and open through the tests. To enhance coherence through each conversation, we instructed the LLM through each phase. Finally, we excluded prompts that had no contribution responses and extra documents that compromised the interview content. The recommended prompt strategy for LLM-supported RTA is presented in Table 2. The main results for each RTA phase are presented in the text and Figure 2.

**Table 2.** Prompt Strategy for LLM Support in RTA.

Recommended prompt strategy for LLM-supported RTA	
System prompt	“You and I are scientists and researchers doing qualitative text analysis using the RTA method. We will analyze the file ‘(name of the attached file)’. You are supposed to use this file to answer my prompts to support my RTA. The text file is an individual interview of one surgical team member’s perspectives on patient safety culture in a surgical context (revise to the relevant type of text, topic, and context).”
1. Familiarization with the dataset	“Support phase 1 in the analysis: Summarize the text’s main repetitive ideas and focus on significant patterns. The summary should be a maximum of 300 words. Do not add any content not present in the file.”
2. Coding	“Support phase 2 in the analysis: A ‘code’ is a phrase of 3–5 words representing a significant topic in the text. Suggest 20 ‘codes’ from the file. Highlight semantic and latent meanings.”
3. Generation of initial themes	“Support phase 3 in the analysis: The next step in this RTA is to merge codes with similar content into 8–10 subthemes. Create 8–10 subthemes with appropriate names, identify which codes you have merged into each subtheme, and describe each of the 8–10 subthemes in 3 sentences.”
4. Development and review of themes	“Support phase 4 in the analysis: Organize subthemes with similar content into 2 or 3 main themes with appropriate names and describe in depth the essence of each theme in 6 sentences.”
5. Refinement, definition, and naming of themes	“Based on this conversation and the file ‘(name of the text file)’, summarize the primary insights and clinical implications for healthcare services.” “Examine the themes you generated (paste in the themes) and subthemes (paste in the subthemes) from the theoretical perspective of (write the relevant phenomenon) presented in the added file ‘name of the attached file.’”
6. Write-up	Attach the text file and prompt in separate conversations: “Critically evaluate the file from the perspective of (write the relevant theoretical framework).” Test with and without attaching a document of the theory. “Suggest theoretical frameworks to which the text file can be related. Use all your information sources. Explain similarities and differences.”

### Familiarization

Through the testing process, the LLM generated increasingly coherent responses, addressed more essential topics from the interview, and made fewer errors. For instance, one of the initial summaries misleadingly emphasized that the side positioning was crucial for reducing patients’ pain. In reality, the interview’s content described this in the context of team communication and adaption. While the summaries occasionally provided a brief impression of the content or highlighted topics that could be further discussed, the LLM consistently excluded essential topics. We tested whether increasing the summary length from 150 to 300 would improve content coverage but that led to more repetitive sentences without added substance. We experienced a risk of attaching documents as they influenced both the summary and the subsequent responses—for example, in Test 5c, where the LLM focused primarily on summarizing the method rather than the interview content. Despite using the similar conversation and system prompt in Tests 6 and 7, the summaries differed, and Test 6 provided the most useful summary, highlighting essential topics such as collaboration, openness, hierarchy, and learning from mistakes. While this aligned with the results of the human analysis, the LLM failed to capture the meaning related to these concepts. The LLM misunderstood one sentence with an ironic undertone and internal humor, revealing a lack of contextual understanding. Relying on these summaries carries a high risk due to potential errors and omitted content. The participant highlighted relationships, trust, and healthcare professionals’ well-being, which the LLM failed to identify. Thus, the LLM did not increase the efficiency of human researchers’ familiarization with the dataset.

### Coding

The LLM’s codes were typically keywords such as “anesthesia” and “surgeon” rather than phrases reflecting meaning. These codes excluded essential content and could overlap highly (e.g., “day shift,” “night shift,” and “sectioned shift”). When prompted to rephrase keywords into ideas of three to five words, the LLM provided dictionary-based synonyms; for example, “teamwork” was changed to “team collaboration.” The codes were mainly semantic and descriptive, even when we prompted the LLM to highlight latent meanings. The LLM provided some potentially useful concepts, such as “feedback culture” and “stress management,” which could contribute to reflexive discussions. Many codes did not contribute to the analyses beyond the summary, and others were errors—for instance, “constructive mistake.” The LLM failed to identify meaningful patterns or latent themes identified in the primary study, such as “authoritarian attitudes as a barrier,” “trust and continuity in relations,” and “building safety by experience.” We experienced a risk of introducing extra documents, as the LLM tended to generate codes based on those documents rather than on the interview file (e.g., “continuous improvement” in Test 5a). These observations indicated that researchers must possess in-depth knowledge of all attached documents and can expect minimal support from the LLM during this phase of analysis.

### Generating Initial Themes

The LLM was prompted to generate themes and subthemes, but provided topic summaries, which were often inappropriately merged. In Tests 6 and 7, the identified themes reflected relevant topics; however, the codes merged in a previous prompt were regenerated, often of content closely related to the generated theme. This may illustrate that an increased number of prompts may widen the distance from the interview text. This distance might resemble a form of abstraction; however, it also introduces the risk of misleading responses. Some themes offered nuances, concepts, or elements for discussions, or challenged the researchers’ preconceptions, for example, by introducing ideas such as “shared decision processes,” “empowerment,” and “speaking up.” The latter two are similar to initial themes from the primary study: “Empowering communication and emphatic leadership” and “Psychological safety in the operating team.” Other themes and subthemes either overlapped with existing ones, were unrelated to the interview data, or lacked substance, such as “focus on safety through positioning and humor.” These results indicate that researchers must apply human reflexivity and maintain a deep knowledge of the data to avoid introducing errors in the analysis. When describing themes and subthemes, the LLM created sentences from the generated codes, added new codes, and reused the same codes across multiple themes. We specified the number of themes or subthemes to guide the output toward our desired outcome.

The LLM altered codes, subthemes, and themes with each conversation, which required us to paste previously generated themes and subthemes into subsequent prompts. For this reason, we merged the prompts for generating and describing themes. We found that increasing the number of prompts or requesting more detail often led to responses that were more misleading or disconnected from the interview data—for instance, the theme “non-pharmacological pain management.” During this phase, we identified repeated content in some outputs and excluded topics altogether. However, the emergence of meaningful and nuanced contributions appeared to be random.

### Developing, Refining, Defining, and Naming Themes

We perceived that the LLM made no contributions to the abstractions of themes and subthemes. The responses often provided synonyms that diverged from the content and context of the interview text. For example, the essential topic “patient positioning” in the operating room was replaced with “ergonomics.” Occasionally, the LLM altered theme names, resulting in new meanings or contexts. The abstraction prompts and their misleading outputs reduced the relevance of subsequent responses. We experienced that increasing the temperature setting, a parameter that controls the variability and creativity of the model’s output, resulted in less relevant, fabricated, and contextually inappropriate responses. Additionally, asking the LLM to identify and describe interactions or overlaps between themes and subthemes did not yield any substantial insights or perspectives. When we requested direct quotes from the interview file, the LLM created fabricated quotes and even referenced page and line numbers. In some cases, these false quotes were entirely unrelated to the themes. Relying on the LLM in this phase poses a risk of incorporating fabricated data into a publication. To mitigate this, we used a default temperature setting of 0.8, aiming for a balance between creativity and consistency. Test 5’s response introduced the relevant topic “adaption in surgical context” in this phase, indicating that the LLMs may surprise, impress, and disappoint in all phases.

### Writing Up

We tested whether the LLM could relate the generated themes to various theories. When we requested suggested theories relevant to the interview text, it provided a mix of appropriate and inappropriate ones. When we introduced specific theories, the LLM could write about them but failed to connect them to the interview texts. Theories introduced by human researchers or suggested by the LLM, with or without attached documents, influenced subsequent responses, making them less inductive and reducing their credibility. For example, when prompting the LLM to relate the interview text to patient safety culture theory, the LLM generated a false definition of patient safety culture and altered the previously generated themes to align with patient safety culture dimensions. In Test 3, when we asked the LLM to examine Donabedian’s theory before patient safety culture theory, the responses focused primarily on Donabedian’s categories of process and structure. In Test 5, both Braun and Clarke’s and Donabedian’s theories were used incorrectly.

In response to an experimental prompt, the LLM claimed to relate geriatric theory to the interview text. The text lacked substance, and the theory influenced the remainder of the conversation. This illustrates that an LLM may provide an answer regardless of its credibility. In some tests, the LLM provided responses suggesting familiarity with the theory. In others, the LLM added information from its training data or generated text that lacked depth and relevance to the theory. In none of these cases did the responses contribute to linking the raw data to the theory or supporting the writing process.

We found that relating the interview text to Donabedian’s theory in a separate conversation yielded more meaningful perspectives than including it within the conversation structured around the analytical process. In some tests, the LLM displayed structural and processual factors from the raw data that aligned with the theory. However, it also included irrelevant and unsubstantial content. Whether or not we attached an additional document about the theory, the responses remained similar. We observed a similar positive effect by examining the interview text in relation to patient safety culture dimensions in a separate conversation. Adding documents to these separate conversations reduced the influence of prior prompts but also obscured the connection to the interview text. In both cases, we prompted the LLM to elaborate after the initial response. These more detailed responses made no contributions to our understanding and often diverged far from the raw data.

Finally, we prompted the LLM to summarize the primary insight from the conversation and suggest potential clinical implications. Some of these summaries captured the broad essence of the interview file. In general, the final summaries from Tests 6 and 7 contributed more effectively to the writing process. They contained fewer errors when we avoided ambiguous prompts and excluded documents that overshadowed the raw data. Nevertheless, the LLM omitted topics or perspectives that we found essential in the primary analysis. The summaries were sometimes repetitive phrases, in which the LLM more confirmed to its own outputs, resulting in a tendency toward self-referential content. The LLM also generated the titles of non-existent publications, requiring that researchers are vigilant about false references. A human researcher with in-depth understanding of the text data is essential to correct LLM errors and reintroduce missing topics and themes. Prompting the LLM for clinical implications produced responses of various quality, from unhelpful or repetitive responses to insights that inspired meaningful discussion. For example, one response emphasized effective coping mechanisms to support organizational learning. In the primary study, this topic was interpreted more specifically in terms of supportive systems after adverse events, interprofessional teams, and participation in learning from cases. The take-home messages from these results are summarized as “Key Takeaways” in Table 3.

**Table 3.** Key Takeaways.

Key takeaways
The recommended prompt strategy may facilitate brainstorming and content for reflexive discussions in random phases
The interview text must be in a language in which the model is trained
Irrelevant information and extra documents may confuse the conversation
The risk of errors and exclusion of essential content requires human reflection and in-depth knowledge of the input and critical examination of the output
Novice and senior researchers/reviewers should engage in the methodological and ethical risks and possibilities in this rapidly developing technology

## Discussion

The overall results of the exploratory testing indicate that RTA currently receives limited support from base LLMs, and LLM-supported RTA is not time-saving compared to traditional qualitative analysis. We suggest that the analysis may require more time due to the need to ensure the quality and accuracy of the responses. Our recommended prompt strategy may serve as a starting point for engaging in LLM-supported conversations and could help reduce the risk of introducing errors into the analysis. We emphasize that human reflexive skills and in-depth knowledge of interview texts, relevant theories, and supplementary documents remain essential for conducting a BigQ RTA, even when supported by foundational LLMs.

### The LLM’s Role in Reflexive Thematic Analysis

The testing showed that responses contributing new perspectives or concepts eligible for reflexive discussions appeared in all phases. However, we emphasize that an LLM cannot replace a human researcher, in-depth understanding, and reflexivity in any single phase. Compared to RTA, which aims to generate semantic and latent codes representing “ideas,” the LLM provided codes as keywords and concepts in these tests (Braun & Clarke, 2022). While other studies report promising results of LLM-supported coding, these outcomes might align more closely with deductive approaches, such as codebook-driven or summative content analysis (Dai et al., 2023; Hitch, 2023; Hsieh & Shannon, 2005; Morgan, 2023; Wachinger et al., 2024). Generating themes in RTA is an active, iterative process of developing interpreted and abstracted themes displaying patterns of meaning in text content (Braun & Clarke, 2022). In our tests, we identified that the LLM often displayed overlapping topics without an overarching coherent meaning story. This is supported by previous studies reporting that LLMs tend to generate descriptive themes (Morgan, 2023; Wachinger et al., 2024). These themes, called “topic summaries,” do not align with the themes according to the RTA approach (Braun & Clarke, 2022). Perkins and Roe (2024) suggested that LLMs may not be suitable for analyzing expressions of latent meaning. LLMs generate texts without inherent meaningfulness, and humans must critically reflect on the sensibility and relevance of machine-generated content (van Manen, 2023). We perceive that these reflections challenge the idea of LLM support in theme generation in RTA. However, LLMs may still support qualitative methods aimed at identifying descriptive and semantic themes (Hsieh & Shannon, 2005). In the context of RTA, we align with previous publications suggesting that LLMs may primarily facilitate brainstorming (Christou, 2023; Owoahene Acheampong & Nyaaba, 2024).

An increasing number of studies describe the role of LLMs in qualitative research (Dai et al., 2023; Paulus & Marone, 2024; Sallam, 2023). After these exploratory tests, we maintain that the LLM is not yet a colleague, assistant, or collaborator in the research loop at this stage of development. The statistical mechanisms by which these tools operate only mimic human intelligence. Accordingly, LLMs cannot be accounted for as authors; only humans can be held accountable for text content (COPE [Committee on Publication Ethics] Council, 2023; van Manen, 2023). However, based on these tests, we endorse that an LLM can be perceived as an uncritical colleague whose feedback must be carefully checked and compared against reliable sources. We suggest that the model produced convincing human-like outcomes that appeared reflective and intellectual, but other random responses without substance appeared immediately after. A previous study argued that, owing to the nature of LLMs, their results can never be fully trusted. They must always be verified by a human; the tools are amoral and not accountable regarding whether responses are true or false (Lindebaum & Fleming, 2024). Rapid text generation is not equivalent to efficient, engaged, and reliable analysis (Paulus & Marone, 2024). Thus, LLMs cannot replace humans’ critical, context-sensitive, and interpretive skills (Christou, 2024).

We perceive that the tests demonstrate that researchers’ human skills and competence are essential to ensuring research quality by evaluating whether the results are grounded in the raw data. The responsibility of methodological quality and ethical considerations lies with the human researcher (Gregor, 2024; Lindebaum & Fleming, 2024). We remain unconvinced that LLMs represent a new paradigm in qualitative research, as suggested by others (Sallam, 2023). The use of LLMs requires a certain level of AI literacy among researchers, including an awareness of how these tools function. Experienced researchers should actively engage in discussions about potential challenges and weaknesses with new colleagues and junior researchers (Hadan et al., 2024; Sallam, 2023).

We will emphasize some of the most crucial considerations when using LLMs to support qualitative analysis. First, we encourage the researcher to obtain technical support to ensure that the LLM responses are based on the whole document and that the chosen model is appropriately embedded according to research data protection and management regulations. Second, to avoid translation errors, we think the model should be trained in the language of the text for analysis. Using models fine-tuned for cross-language understanding or implementing translation methods that preserve context might yield better results than direct translation (Li et al., 2024). Third, we suggest that there is a risk of misleading prompts and extra documents as well as and also a risk of self-referential text based on repetitive tendencies and text generation based on the LLM’s responses throughout the conversation. Finally, we consider that the structure and functions of LLMs require an assessment of whether the responses are true and reliable.

The linguistic predictability inherent in LLM algorithms, combined with their acontextual nature regarding personal, social, and historical conditions, leads to high-probability choices when generating texts to support qualitative analysis (Lindebaum & Fleming, 2024). This stands in contrast to BigQ researchers, who, through reflexivity and contextual understanding, deliberately explore low-probability choices and alternative explanations to deepen the understanding of a phenomenon (Braun & Clarke, 2022; Lindebaum & Fleming, 2024). We highlight this contrast as indicative of potential epistemological limitations in integrating LLMs into BigQ research.

### Epistemological and Methodological Considerations

We will raise fundamental questions about epistemological and methodological coherence and trustworthiness in embracing the innovative technology of LLMs within the interpretative paradigm (Kuhn, 1996). RTA often relies on an ontological view rooted in relativist perspectives, emphasizing the role of human interpretations. It may also align with critical realism, which positions itself between the relativistic and mind-independent views of realism (Braun & Clarke, 2013). The ontological view is connected to the epistemological perspective on what constitutes legitimated knowledge and how valid knowledge can be generated (Braun & Clarke, 2013). In BigQ research, valid and meaningful knowledge is often generated through a comprehensive, engaged, and collaborative process that values qualitative principles such as subjectivity and reflexivity (Braun & Clarke, 2013). RTA emphasizes reflexive memos and critical discussions as an iterative process between the phases and between interpretations and raw data (Braun & Clarke, 2006, 2022). This requires a trusting collaborative research panel that allows critical and challenging questions and actively generates abstract themes over an extended time frame (Braun & Clarke, 2022). With this philosophy of science background, we think that there are reasons to be critical about whether BigQ may be consistent with LLM support.

Methodological integrity and quality in qualitative research are often discussed by using Lincoln and Guba’s framework for trustworthiness (Polit & Beck, 2021). In RTA, the quality criterion *confirmability* refers to generating themes representing the participants’ voices and perspectives rather than a congruence of objective truth (Lincoln et al., 1985; Polit & Beck, 2021). We experienced that the LLM excluded essential content and included false content through the tests, indicating that the human researcher must ensure confirmability in qualitative analysis. Our worst example of threatened confirmability was that the LLM created false participant quotes, making it an untrustworthy tool and requiring humans to complete their familiarization phase thoroughly.

Demonstrating *credibility* in LLM-supported RTA requires a transparent audit trail of the process to establish confidence (Polit & Beck, 2021). We suggest that the rapid and conclusive responses from an LLM challenge the researcher to explain what contributions it made at which stage, how its responses influenced the process, and why its contributions were relevant to the analysis. We may challenge this critical argument owing to the need for the same descriptions from an analysis panel discussion with various subjective researchers. We suggest an analysis panel promoting human values, reflexivity, and contextual understanding to increase credibility compared to a black-box answer. Sallam (2023) supported this by describing this black-box application as a transparency issue. This issue implies a lack of an explanation of how content is generated, its sources, and its level of accuracy and accountability (Gregor, 2024; Ray, 2023; Sallam, 2023). This challenges the possibility of transparent reporting, and we think that the various responses over time also complicate the *dependability* and replication of the analysis in methods in which transparency is valued. The quality criterion *transferability*, referring to whether results can be transferred to other contexts, also requires detailed descriptions of the generated results, which is challenging because of the text-generating black box (Polit & Beck, 2021).

*Authenticity* is a quality criterion that refers to the authentic feelings and sensitivity to participants’ emotions, lives, and contexts when reading the research (Polit & Beck, 2021). Sensitivity may be described as a curious and critical approach to life as well as the knowledge and ability to step outside one’s values and assumptions to gain a deeper understanding of research data (Braun & Clarke, 2013). When we consider the many unsubstantiated responses during the testing and base these on the knowledge of how LLMs produce their outcomes, we find it difficult to believe that a machine can produce results close to such understandings. LLMs do not reflect on reality and whether the text is true or false and thus whether it is authentic (Lindebaum & Fleming, 2024). Human insights and reflections are necessary to evaluate the trustworthiness of LLMs’ responses to interview texts and to write detailed audit trails (Polit & Beck, 2021). We consider human reflexive qualities essential for preserving methodological integrity. Therefore, from our point of view and at this point in technological development, LLMs might be redundant in BigQ research. Consequently, we are inclined to support a previous paper that placed LLMs in the realm of pseudo-science (Lindebaum & Fleming, 2024).

### Ethical Considerations

During this study, we faced several ethical challenges. The first was the privacy and data protection of the participants in the primary study. The interview data were unidentified but not anonymous. Sharing data with an online LLM raises ethical concerns because commercial actors may use the input for further training and development of the quality of their profiled products (Davison et al., 2024). The European Union’s General Data Protection Regulation protects the privacy of research participants by ensuring that their data are used only for the purposes described in the information and consent form (European Union, 2016). This means that the responsible researcher is generally not permitted to insert personal data into an online LLM that stores or uses the information for training. For example, ChatGPT stores and uses content to train its models unless users actively turn this option off in their settings (OpenAI, 2025). Hence, researchers should not insert interview data into online LLMs without ensuring that the data are properly protected, which often requires licenses that involve payment (OpenAI, 2025) or offline platforms that use powerful computers (LM Studio, 2025; Ollama, 2025).

Another ethical concern we perceived was that research in health science often has clinical implications and may impact healthcare performance. Healthcare professionals are raised under the Hippocratic Oath and the medical priority of “Primus non nocere” (“First, do no harm”) (National Library of Medicine, 2025). The United Nations Educational, Scientific and Cultural Organization’s (UNESCO) recommendations on the ethics of AI highlight that it should harm no human beings or communities (UNESCO Digital Library, 2021). We will highlight that this study displayed multiple aspects indicating that responses should be cautiously trusted to avoid harm. LLMs create responses on linguistic predictability without commitment and responsibility, which can lead to incorrect information (Gregor, 2024; Lindebaum & Fleming, 2024; OpenAI, 2025). Owing to the increased quality and perceived credibility of LLM responses, errors become challenging to identify, and the risk of taking false facts into the analysis increases (Ji et al., 2023). We emphasize that including LLMs’ errors in qualitative analysis in general must be avoided. In health science, this may, at worst, harm patients. The issue of errors could be mitigated by implementing techniques such as RAG with stronger grounding, self-consistency checking, and uncertainty estimation approaches (Koc et al., 2024; Niu et al., 2024).

The LLM used in this study generated responses based on the files that we attached to the conversations. However, we identified that this LLM also retrieved information from other sources it was trained on. From a critical perspective, LLMs can be trained in published studies and influence their responses to align them with previous research (Marshall & Naff, 2024). Moreover, LLM output can be improved by RAG implementation using techniques that enforce source attribution and fine-tune the model specifically for document-grounded analysis (Koc et al., 2024; Niu et al., 2024). Independent of temperature setting, the LLM can produce errors, as demonstrated in our tests with the default temperature (Renze, 2024). The sources in which trained LLMs can reflect preconceptions and attitudes regarding gender, power, and professionals in their responses are often influenced by Western culture (Davison et al., 2024). We were unable to identify this issue during our testing process. However, this could constitute a risk of errors and a threat to trustworthiness in a single study. We raise the concern that from a broader perspective, this phenomenon might pose a threat to the body of knowledge, values, and attitudes within the research communities. An illustrative example from our testing could be the LLM’s promotion of the benefits of the side position for surgical patients. At worst, this might generate misleading evidence synthesized in reviews as guidance for clinical changes. The ethical concerns regarding LLMs in qualitative research align with the limited utilization of LLM-supported decision-making in clinical work; LLMs are influenced by training data and may harm patients (Park et al., 2024). From a utilitarian perspective, these consequences may not be compared to the possible benefits of more efficient processes (Rosen, 2003).

### Recommendations for Further Research

A large dataset from real-life health science research, that has not yet been analyzed, should be tested using the recommended strategy. This should include comparison with a parallel traditionally conducted RTA as part of a qualitative blinded testing design. Additionally, the strategy could be tested across diverse types of qualitative data to assess its generalizability. Future research should also apply our proposed testing framework to more advanced AI systems, including models with sophisticated reasoning and “deep research” capabilities. A comparative analysis would be invaluable in determining whether these newer technologies can overcome the limitations in interpretation and abstraction identified in our study. Finally, future research should explore the potential of LLM to support collaborative analysis across domains and multimodal approaches.

### Strengths and Limitations

This study has multiple strengths. First, the selected interview content is prominent in the primary study’s results, increasing the analysis panel’s ability to evaluate the trustworthiness of the LLM-generated responses. Second, two authors had in-depth insight into the interview text and the attached documents, enabling them to evaluate whether the responses were grounded in the raw data, influenced by added documents, or derived from other information sources. However, this insight might also represent a limitation when compared to a more inductive approach in this exploratory design. A third strength lies in the ethical handling of privacy and data protection, ensured by using an offline LLM. This offline model may also present a limitation due to its embeddings and parameters. A larger model might offer greater support for RTA than the LLM Mistral-7B used in this study. Another limitation concerns the study’s transferability, given the rapid pace of technological development. This study does not evaluate the performance of more recent models equipped with advanced multi-step reasoning (e.g., chain-of-thought) or agentic “deep research” capabilities, which were nascent or not available in secure, offline platforms at the time of analysis (Deepseek AI, 2025). Nevertheless, the epistemological challenges we identify related to subjectivity, reflexivity, and authentic meaning-making are likely to persist. This study can therefore contribute to researchers’ reflections, insights, and engagement in LLMs in BigQ research.

## Conclusion

Currently, base LLMs do not reduce the time researchers spend on analytical work in RTA. However, with a clarified prompt strategy and human-led familiarization, this class of LLMs may contribute concepts and nuances for reflection within an analysis panel and potentially uncover gaps in researchers’ perspectives. Several challenges remain, including technical aspects such as data protection and selecting the most appropriate model. Researchers should also be aware of issues related to translation, confusing prompts and documents, self-referential text, and errors. Integrating LLM-generated responses into analysis may compromise the trustworthiness of results and threaten researchers’ methodological and epistemological integrity. These concerns also raise ethical questions, particularly given the potential clinical implication in health science research. We suggest that LLM-supported RTA requires human reflexivity and in-depth familiarity with the raw data, relevant theories, and any supplementary documents. This raises a more fundamental question: Should we aim to make the analytical process more efficient, or is this one of a qualitative researcher’s most essential tasks? As a final remark, the quality of the outcomes produced by LLMs does not currently rival the work of qualitative researchers or their role in generating knowledge. However, LLMs may pose a threat to qualitative research if used uncritically, especially if researchers overlook their limitations, underestimate the risk involved, or fail to keep pace with their rapid development and evolving capabilities. Senior researchers and peer reviewers should acquire in-depth knowledge of LLMs to recognize LLM-generated work and to effectively supervise and support junior researchers.

## Supplemental Material

Supplemental Material - Reflecting on LLM Support in Reflexive Thematic Analysis: An Explorative Study Supplemental Material for Reflecting on LLM Support in Reflexive Thematic Analysis: An Explorative Study by Magnhild Vikan, Ramtin Aryan, Mari Serine Kannelønning, Michael Alexander Riegler, and Stein Ove Danielsen in Qualitative Health Research
