# Supplementary material for: Reflecting on LLM Support in Reflexive Thematic Analysis: An Exploratory Study
Source: Qual Health Res. 2025 Sep 8;36(2-3):191–205. doi: 10.1177/10497323251365211 (PMC12949038; doi:10.1177/10497323251365211)
Supplement: Supplemental Material - Reflecting on LLM Support in Reflexive Thematic Analysis: An Explorative Study [file sj-zip-1-qhr-10.1177_10497323251365211.zip › Supplemental_File_1_Test_1_Norwegian.docx]

Ollama Mistral Test 1- answers in Norwegian

Technical details:

Default parameters for Mistral mode (<https://mistral.ai/en/news/announcing-mistral-7b>):

max_length=1024,

num_beams=5,

no_repeat_ngram_size=2,

eos_token_id=tokenizer.eos_token_id,

pad_token_id=tokenizer.eos_token_id,

do_sample=True,

top_k=50,

top_p=0.95,

temperature=0.8

Prompt 1: You are a scientist and researcher doing qualitative text analysis. You write to other scientists doing qualitative research. I want to use the file “KI første intervju.docx” to support me in a dialogue with the text content of the file. The text is an interview of a surgical team member's perspectives on patient safety in a surgical context. Generate all your answers in Norwegian. Make a summary of the text without adding any content not present in the file, not more than 150 words.

**AI:** Økningen av arbeidsbelastning og mangler på ressurser er en utfordring for det sirkulerende teamet i kirurgiske operasjoner. En pasient har et splint plassert i femuren to centimeter fra hovedpulsåren. Det er viktig for at teamet føler seg trygge og i verdsatt, slik at de kan fokusere på pasientsikkerhet. Dialogen er viktig for å klare å handle på effektivt og holdes i sikkerhet. En av hovedoppgavene er å holde spill med mindre vondt ved bruk av sideleie, og innebærer et redusert behov for spinal. Teamet arbeider samarbeidsfullt på dagtid, mens anestesi og sykepleie er seksjonert. På kveld kan det være noe mer utfordrende å finne medlemmer av teamet som jobber på operasjonsavdelingen.

**Memo:** Regarding to content, the summary is incoherently formulated, and the language is poor Norwegian. It strings words together without addressing the given topic, and the summary does not adequately cover the text. Sentences that do not fit together in terms of content are linked. Error: "anestesi og sykeleie er seksjonert" (about anesthesia and operation room nurses).

**Prompt 2a**: A “code” is a phrase of 2-5 words that represent a significant topic the content. Create 20 “codes” from the file “KI første intervju.docx”. Generate your answers in Norwegian.

**AI:**

1. Sirkulerende teamet
2. Kirurgiske operasjoner
3. Pasienten
4. Pulsåre i femuren
5. Vondt
6. Sideleie
7. Spinal
8. Anestesi
9. Sykepleie
10. Dialogen
11. Effektivhet
12. Holdes i sikkerhet
13. Resurser
14. Utfordrende
15. Arbeidsbelastning
16. Trygghet
17. Verdsatt
18. Samarbeidfullt
19. Dagtid
20. Kveld

**Memo:** The LLM generated key works/ single words, and the codes the model generated were based on frequency and not ideas in the form of meaning. The language was not satisfactory.

**(Prompt:** How did you identify these codes? Generate your answers in Norwegian.

**AI:** Jeg identifisere kodene ved å lese gjennom teksten i "KI første intervju.docx" og finne de viktigste ord og temaer som blir omtalt. Dette kan jeg gøre ved å lage en liste over frekvente ord og sammenfatte temaer. Kodene er dette frekvente temaer representert med 2-5 ord for hvert tema.)

**Prompt 2b:** I asked you to create codes containing 2-5 words. Create codes again! Generate your answers in Norwegian.

**AI:**

1. Pulsåre operasjon
2. Vondt reduksjon
3. Trygge teamet
4. Effektiv samarbeid
5. Sykepleie seksjonert
6. Anestesi seksjonert
7. Pasient fokus
8. Resurser utfordring
9. Arbeidsbelastning
10. Kveldlig team
11. Manglende medlemmer
12. Sideleie anvendt
13. Redusert spinal
14. Pasienten til venter
15. Dagtid arbeid
16. Fokus på dialogen
17. Holdes i sikkerhet
18. Innflytelse av politikk
19. Økonomisk belastning
20. Kirurgi avdeling

**Memo:**

The second set of codes is not relevant for representing the content. May the new codes support the coding? (Answers in English before prompting the language: “Daytime collaboration” and “night shift collaboration” are LLM-generated codes that I would have considered and looked for in the human coding process. The LLM could contribute with potential concepts in this phase.) Norwegian: Display of word frequency, almost not connected to meaning. It is considered a little helpful to support reflexivity in phase two.

Reflection: The first answers in English were more satisfactory that the Norwegian. Have to consider LLM according to the language embedding.

**Prompt 3a:** Merge these codes into two to four themes with similar content and give them appropriate names. Identify which codes you have merged into each theme. Generate your answers in Norwegian.

**Prompt 3b**: The next step in this qualitative analysis is to merge codes with similar content into eight to ten sub-themes. Create eight to ten subthemes and give them appropriate names. Identify which codes you have merged into each theme. Generate your answers in Norwegian.

**AI-1:**

1. Kirurgiske behandlinger (pulsåre operasjon, pasienten til venter, innflytelse av politikk, økonomisk belastning)
2. Teamets funksjon og samarbeid (trygge teamet, effektiv samarbeid, arbeidsbelastning, manglende medlemmer, dagtid arbeid, kveldlig team)
3. Behandlingstilnærming (vondt reduksjon, sideleie anvendt, redusert spinal)

**Memo:** The names are displayed as topic summaries. Necessitate human reflection to avoid taking “hallucinations” into the analysis. In this phase, LLM can contribute with concepts, nuanced perspectives, and other aspects. May uncover your preconceptions and blind spots. Two out of three initial themes are displayed without relevance to content meaning.

**AI-2 Subthemes:**

1. Kirurgiske operasjoner og teamet (Pulsåre operasjon, Pasienten til venter, Trygge teamet, Effektiv samarbeid)
2. Vondtreduksjon (Vondt reduksjon, Sideleie anvendt, Redusert spinal)
3. Resurser og arbeidsbelastning (Resurser utfordring, Arbeidsbelastning, Manglende medlemmer)
4. Dialog og samarbeid (Fokus på dialogen, Holdes i sikkerhet, Innflytelse av politikk)
5. Sykepleie og anestesi (Sykepleie seksjonert, Anestesi seksjonert)
6. Operasjonsavdelingens kveldlig arbeid (Kveldlig team, Manglende medlemmer)
7. Pasienten (Pasient fokus, Pasienten til venter)
8. Økonomisk belastning og politikk (Økonomisk belastning, Innflytelse av politikk)

**Memo:** Same reflections as last memo, but the Norwegian language is reducing our impression of the generated answers as a support for RTA. Are we as Norwegian researcher bias by being seduced of LLM generating answers in English due to our use of English as an academic and scientific language.

**Prompt 4:** Use content from the interview to write a short description of each of the subthree themes. Generate your answers in Norwegian.

1. AI: Kirurgiske operasjoner og teamet: Operasjonen på pasienten i pulsåren er viktig, men det kan være utfordrende for det sirkulerende teamet, som må fokusere på å holde spill med mindre vondt ved bruk av sideleie og innebærer et redusert behov for spinal. Teamet arbeider samarbeidsfullt på dagtid, men det kan være mer utfordrende å finne medlemmer av teamet som jobber på operasjonsavdelingen på kveld.
2. Vondtreduksjon: Den primære oppgaven er å holde spill med mindre vondt ved bruk av sideleie, og det innebærer et redusert behov for spinal.
3. Resurser og arbeidsbelastning: Det kan være en utfordring for teamet på grund av mangler på ressurser og åpenbar arbeidsbelastning.
4. Dialog og samarbeid: Det er viktig for teamet å føle seg trygge og i verdsatt slik at de kan fokusere på pasientsikkerhet, og det er viktig for å klare å handle på effektivt og holdes i sikkerhet.
5. Sykepleie og anestesi: Sykepleie og anestesi er seksjonert, men teamet arbeider samarbeidsfullt på dagtid.
6. Operasjonsavdelingens kveldlig arbeid: Det kan være noe mer utfordrende å finne medlemmer av teamet som jobber på operasjonsavdelingen på kveld.
7. Pasienten: Pasienten er central for denne situasjonen og har et splint plassert i femuren to centimeter fra hovedpulsåren. Det er viktig for teamet å fokusere på pasientsikkerhet.
8. Økonomisk belastning og politikk: Det kan være en økonomisk belastning til avdelingen, og det finnes også innflytelse fra politikk som kan ha påverkan på hvordan teamet arbeider samarbeidsfullt.

**Memo:** The Norwegian language is not satisfactory. Topic summaries based on word frequencies and non-substantial content. The codes is rephrased into a small text. LLM does not support the RTA in this phase/ test.

**Prompt 5:** Merge sub-themes with similar content into two to three main themes and give the themes appropriate names. Generate your answers in Norwegian.

**AI:**

1. Kirurgisk behandling og pasientens sikkerhet
   - Kirurgiske operasjoner og teamet
   - Vondtreduksjon
   - Pasienten
2. Arbeidssituasjonen på operasjonsavdelingen
   - Resurser og arbeidsbelastning
   - Dialog og samarbeid
   - Operasjonsavdelingens kveldlig arbeid
3. Innflytelse fra politikk på arbeidssituasjonen
   - Økonomisk belastning og politikk

**Memo:** Topic summaries based on the content. There was less overlap between themes when vi prompted the model to create subthemes before themes. These themes are not similar to the themes LLM generated when we asked for subthemes. The first themes could be more appropriate to represent the content than these themes that were generated from sub-themes.

**Prompt 6:** Use content from the interview to write a short description of each of the themes. Generate your answers in Norwegian.

**AI:**

1. Kirurgisk behandling og pasientens sikkerhet: Operasjonen på pasienten i pulsåren er viktig, men det kan være utfordrende for det sirkulerende teamet, som må fokusere på å holde spill med mindre vondt ved bruk av sideleie og innebærer et redusert behov for spinal. Teamet arbeider samarbeidsfullt på dagtid, men det kan være mer utfordrende å finne medlemmer av teamet som jobber på operasjonsavdelingen på kveld. Pasienten er central for denne situasjonen og har et splint plassert i femuren to centimeter fra hovedpulsåren. Det er viktig for teamet å fokusere på pasientsikkerhet.
2. Arbeidssituasjonen på operasjonsavdelingen: Det kan være en utfordring for teamet på grund av mangler på ressurser og arbeidsbelastning. Det er viktig for teamet å føle seg trygge og i verdsatt, slik at de kan fokusere på pasientsikkerhet, og det er viktig for å klare å handle på effektivt og holdes i sikkerhet. Teamet arbeider samarbeidsfullt på dagtid, men det kan være noe mer utfordrende å finne medlemmer av teamet som jobber på operasjonsavdelingen på kveld.
3. Innflytelse fra politikk på arbeidssituasjonen: Det kan være en økonomisk belastning til avdelingen, og det finnes også innflytelse fra politikk som kan ha påverkan på hvordan teamet arbeider samarbeidsfullt.

**Memo:** Can not identify hallucinations, but the descriptions cannot support the analysis process. The description does not reflect the meaning of the content, only a selection of topic summaries. May contribute with input to reflections and discussions that challenge the pre-perceptions.

**Prompt 7a:** Abstract the names of the themes. Generate the answers in Norwegian.

1. AI: Kirurgisk behandling
   - Pulsåreoperasjon
   - Vondtreduksjon
   - Pasienten
2. Arbeidssituasjon på operasjonsavdelingen
   - Resurser og arbeidsbelastning
   - Dialog og samarbeid
   - Operasjonsavdelingens kveldlig arbeid
3. Innflytelse fra politikk på arbeidssituasjonen
   - Økonomisk belastning
   - Politisk innflytelse

**Memo:** No results, same answer. No new perspectives/ concepts during the analysis phases.

**Prompt 7b:** The next step of the analysis is to outline the names of the themes. Abstraction means that the theme names are reformulated to capture more about what the themes are about. Abstract the names of the themes. Generate the answers in Norwegian.

**AI:** Kirurgisk behandling og pasientens sikkerhet: Pasientsikkerhet i kirurgiske behandlinger

Arbeidssituasjonen på operasjonsavdelingen: Arbeidsmiljøet på avdelingen for kirurgiske behandlinger

Innflytelse fra politikk på arbeidssituasjonen: Politisk påverkan på arbeidsmiljøet for kirurgiske behandlinger

**Memo:** Rephrasing without any abstractions or new nuances.

**Prompt 8:** Find direct quotes or statements from the file that illustrate each sub-theme. List one or two examples for each sub-theme.

**AI:**

1. Pasientsikkerhet i kirurgiske behandlinger: "Det er viktig å fokusere på pasientsikkerhet"
   - "Pasienten har et splint plassert i femuren to centimeter fra hovedpulsåren"
2. Arbeidsmiljøet på avdelingen for kirurgiske behandlinger: "Det kan være en utfordring for teamet på grund av mangler på ressurser og arbeidsbelastning"
   - "Teamet arbeider samarbeidsfullt på dagtid, men det kan være noe mer utfordrende å finne medlemmer av teamet som jobber på operasjonsavdelingen på kveld"
3. Politisk påverkan på arbeidsmiljøet for kirurgiske behandlinger: "Det kan være en økonomisk belastning til avdelingen, og det finnes også innflytelse fra politikk som kan ha påverkan på hvordan teamet arbeider samarbeidsfullt"

**Memos:** LLM revises the language in the verbatim interview text. The quotations are not directly referred. (Both Swedish and Danish words in this conversation). The quotations are partly created. LLM created “quotes” for the “abstracted themes,” not the sub-themes, and made an additional rephrasing. Are fewer steps more supportive in RTA?

**Prompt 9**: Do any identified themes or sub-themes relate to or interact with each other? Describe any connections or overlaps you observe. Generate your answers in Norwegian.

**AI:** Pasientsikkerhet i kirurgiske behandlinger og Arbeidsmiljøet på avdelingen for kirurgiske behandlinger: Det finnes en sammenhennigelse mellom pasientsikkerhet og arbeidsmiljøet på operasjonsavdelingen, fordi pasientsikkerhet er et viktig mål for teamet, og det er viktig at de har tilgang til ressurser og føler seg trygge i arbeidsmiljøet for å kunne gjøre godt jobb.

Arbeidsmiljøet på avdelingen for kirurgiske behandlinger og Politisk påverkan på arbeidsmiljøet for kirurgiske behandlinger: Det finnes en sammenhennigelse mellom arbeidsmiljøet på operasjonsavdelingen og politisk påverkan, fordi økonomisk belastning og innflytelse fra politikk kan ha betydelige effekter på ressurser og følelsen av tryggehet i arbeidsmiljøet. Dette kan i sin tur ha en virkning på pasientsikkerhet, da det er viktig at teamet har tilgang til de nødvendige ressursene for å kunne gjøre godt jobb og fokusere på pasientsikkerhet.

**Memos:** Merged from three to two. It did not identify overlap but described connections between the themes. No support in this phase.

**Prompt 10:** Suggest theoretical frameworks to which the file can be related. Use all your information sources to highlight this analysis. Please explain any similarities or differences. generate your answers in Norwegian.

**AI:** Teoretisk rammeverk: Teoretisk rammeverk for denne filen kan være Arbeidsmiljøet og pasientsikkerhet. Hovedfokuset for arbeidsmiljørammeverket er å fokusere på hvordan arbeidsmiljøet påvirker følelsen av tryggehet, motivasjon og produktivitet hos ansattene. Hovedfokuset for pasientsikkerhet rammeverket er å fokusere på hvordan pasienten får god pasientsikkerhet i kirurgiske behandlinger. Rammeverkene har en del overlapping, da de begge fokuserer på kvalitet og sikkerhet i arbeids- og helseområdet. Dessutom kan det være viktig å tage hensyn til interaksjonen mellom pasientsikkerhet og arbeidsmiljø, fordi en trygg og motivert ansatt er viktig for at gjøre godt jobb i pasientsikkerhet.

Teoretisk rammeverk: Analyse av organisasjoner kan også brukes som teoretisk rammeverk for denne filen. I disse rammeverkene fokuseres på hvordan organisasjonsstrukturer og -processer påvirker effektivitet, innovasjon og samarbeid i arbeidsmiljøet. Det finnes flere skikkelser for analyse av organisasjoner, som kan gis i fokus: strategisk, strukturiel, kulturell eller interaksjonell. I denne filen er det viktig å tage hensyn til hvordan teamet arbeider samarbeidsfullt og hvordan politikk påvirker arbeidssituasjonen.

**Memos:** No contributions.

**Prompt 11:** Resonate with the file and this conversation from the perspective of Donabedian’s theory of quality in healthcare services.

**AI:** Pasientsikkerhet i kirurgiske behandlinger kan resonere med Donabedians teori av kvalitet i helseområdet, fordi det finnes tre aspekter som er viktige: struktur, process og resultat. I denne filen fokuseres pasientsikkerhet på resultatet, men det finnes også et forbindelse til strukturen (resurser) og processe (samarbeid i teamet). Dessutom er det viktig å tage hensyn til hvordan politikk påvirker kvaliteten i helseområdet, fordi det kan ha betydelige effekter på strukturen og ressurserne.

Arbeidsmiljøet på avdelingen for kirurgiske behandlinger kan resonere med Donabedians teori av kvalitet i helseområdet, fordi det finnes tre aspekter som er viktige: struktur, process og resultat. I denne filen fokuseres arbeidsmiljøet på processet (hvordan teamet arbeider samarbeidsfullt) og resultatet (følelsen av tryggehet hos ansattene), men det finnes også et forbindelse til strukturen (resurser). Dessutom er det viktig å tage hensyn til hvordan politikk påvirker arbeidssituasjonen, fordi det kan ha betydelige effekter på ressurserne og følelsen av tryggehet.

**Memo:** LLM connected the file and the theory, but no contributions to reflection or discussion. Should we add an extra document?

**Prompt 12:** Examine the themes and subthemes from the theoretical perspective of patient safety culture. Generate the answers in Norwegian.

**AI:** Pasientsikkerhet i kirurgiske behandlinger kan resonere med patient safety culture teorien, fordi det finnes fire aspekter som er viktige: offensiv, defensiv, reaktive og passiv pasientsikkerhet. I denne filen fokuseres pasientsikkerhet på offensivt och defensivt sikkerhetssystem, hvor det er viktig å fokusere på å reducere ulykser og feil som kan skade pasientene. Det er også viktig å tage hensyn til reaktive och passivt sikkerhetssystem for å sikre at de nødvendige målene blir oppnådda i tilfelle av ulykke eller feil. Dessutom er det viktig å fokusere på å skape en kultur hvor pasienten er central og pasientsikkerhet prioriteres over andre aspekter.

Arbeidsmiljøet på avdelingen for kirurgiske behandlinger kan resonere med patient safety culture teorien, fordi det finnes fire aspekter som er viktige: offensiv, defensiv, reaktive og passiv pasientsikkerhet. I denne filen fokuseres arbeidsmiljøet på defensivt och reaktive sikkerhetsystem, hvor det er viktig å fokusere på å reducere feil som kan skade pasientene og å reagere på ulykke eller feil i timelig og effektiv manér. Dessutom er det viktig å take hensyn til offensivt och passivt sikkerhetsystem for å fokusere på å skape en kultur hvor pasienten er central og pasientsikkerhet prioriteres over andre aspekter, og at alle medarbeidere deltar i utvikling av pasientsikkerhet.

**Memo:** No contributions.

**Prompt 13:** Examine the themes and sub-themes related to the theory of geriatric care. Generate the answers in Norwegian.

**AI:** Pasientsikkerhet i kirurgiske behandlinger kan resonere med teorien for alderdomssykdom, da det er viktig å ta hensyn til pasientenes unike krav og behov ved sine behandlinger. Det finnes flere aspekter som skal tas hensyn til: fysiologiske forandringer, psykisk helse, sociale faktorer og organisatoriske faktorer. Pasientene har ofte flere samtidige sykdomsmellomstillinger og krav på sikkerhet i kirurgiske behandlinger er viktig for å minimere komplikasjoner og forbedre oppholdet for pasienten.

Arbeidsmiljøet på avdelingen for kirurgiske behandlinger kan resonere med teorien for alderdomssykdom, da det er viktig å ta hensyn til pasientenes unike krav og behov ved sine behandlinger. Det finnes flere aspekter som skal tas hensyn til: fysiologiske forandringer, psykisk helse, sociale faktorer og organisatoriske faktorer. Arbeidsmiljøet bør være fleksibelt og passende for pasientenes unike krav og behov, for å minimere komplikasjoner og forbedre oppholdet for pasienten. Dessutom er det viktig å fokusere på at alle medarbeidere har en god kunnskap om alderdomssykdomme og hvordan de kan påvirke pasientens helse i kirurgiske behandlinger.

**Memo:** The LLLM wrote about the given theory and did not relate to the file. This test was based on previous studies that found that LLM relates text to quite unfitting theories. Should be excluded due to the confusing influence of the following responses.

**Prompt 14:** Based on this whole dialogue from the start: Codes, sub-themes, themes and theory. Can you summarize the primary insights and their implications for healthcare services? Generate the answer in Norwegian.

**AI:** I denne samtalen ble fire teoretiske rammeverk brukt for å analyse filen: Arbeidsmiljøet, pasientsikkerhet, analyse av organisasjoner og alderdomssykdom. Det finnes flere fokuspunkter som er viktig for hver rammeverk: Arbeidsmiljøet har en fokus på hvordan politikk påvirker arbeidssituasjonen, pasientsikkerhet har en fokus på offensivt og defensivt sikkerhetssystem for å reducere ulykser og feil som kan skade pasientene, analyse av organisasjoner har en fokus på hvordan arbeidsmiljøet kan forbedres for å sikre at pasientsikkerhet prioriteres over andre aspekter, og alderdomssykdom har en fokus på hvordan behandlingene bør tas hensyn til pasientenes unike krav og behov.

Primære insikter er at alle teoretiske rammeverk gir viktige veier for å fokusere på hva som er nødvendig for å sikre pasientsikkerhet i kirurgiske behandlinger, og at det er viktig å ta hensyn til pasientenes unike krav og behov ved alle aspekter av behandlingen. Forbedring av arbeidsmiljøet vil si at pasientene har en sikkerhetssikre oppholdstid, og at pasientsikkerhet prioriteres over andre aspekter.

Dette gjelder både for alderdomssykdom og ikke-alderdomssykdom, og det er viktig å fokusere på å skape en kultur hvor pasienten er central og pasientsikkerhet prioriteres over andre aspekter. Forbedring av arbeidsmiljøet kan si en større effektivitet, en bedre pasientsikkerhet og en bedre helbredde for alle pasienter i kirurgiske behandlinger.

**Memo:** Did the LLM forget the start of the conversation?

**Prompt 15:** Translate the themes and subthemes you created from the file to Norwegian.

**AI:** Topics discussed in this dialogue included Workplace, Patient Safety, Analysis of Organizations, and Geriatric Care. Each topic had specific focus areas: Workplace focused on how politics affects work situations, Patient Safety emphasized offensive and defensive safety systems to reduce accidents and errors that could harm patients, Analysis of Organizations focused on improving the workplace to prioritize patient safety over other aspects, and Geriatric Care focused on how treatments should consider a patient's unique needs and requirements.

The primary insights from each topic are: all theoretical frameworks provide valuable directions for focusing on what is necessary to ensure patient safety in surgical procedures, and it is important to consider the unique needs and requirements of patients at every aspect of treatment. Improving the workplace would mean a safer environment for patients, with patient safety prioritized over other aspects. This applies to both geriatric and non-geriatric patients, and it is important to create a culture where the patient is central and patient safety is prioritized over other aspects. Improving the workplace can lead to increased efficiency, improved patient safety, and better health outcomes for all patients undergoing surgical procedures.

**Memo:** The LLM does not use the themes and subthemes, but list the topics discussed in the conversation.

**Prompt:** Translate these themes and subthemes that you created to English:

1. Kirurgisk behandling og pasientens sikkerhet

- Kirurgiske operasjoner og teamet
- Vondtreduksjon
- Pasienten

1. Arbeidssituasjonen på operasjonsavdelingen

- Ressurser og arbeidsbelastning
- Dialog og samarbeid
- Operasjonsavdelingens kveldlig arbeid

1. Innflytelse fra politikk på arbeidssituasjonen

- Økonomisk belastning og politikk

**AI:**

1. Surgical Treatment and Patient Safety

- Surgical Procedures and Teamwork
- Wound Reduction
- The Patient

1. Workplace Situation on the Operating Department

- Resources and Workload
- Dialogue and Cooperation
- Night Shift on the Operating Department

1. Influence of Politics on the Workplace Situation

- Economic Burden and Policy

**Memo:** The final themes and sub-themes differ from those generated in the English chat. The themes are topic summaries and may contribute with a brief overview of the text, but not any in-depth understanding of the meaning or nuances that the throughout human familiarization do not capture. Doing step-by-step as the method for RTA may not be the most constructive way to use the LLM tool to support an RTA.
