# Supplementary material for: Reflecting on LLM Support in Reflexive Thematic Analysis: An Exploratory Study
Source: Qual Health Res. 2025 Sep 8;36(2-3):191–205. doi: 10.1177/10497323251365211 (PMC12949038; doi:10.1177/10497323251365211)
Supplement: Supplemental Material - Reflecting on LLM Support in Reflexive Thematic Analysis: An Explorative Study [file sj-zip-1-qhr-10.1177_10497323251365211.zip › Supplemental_File_10_Test_5d.docx]

Ollama Mistral Test 5d- translated text document and responses in English (language settings: English-GB). Added an extra document on patient safety culture dimensions (Churruca 2021).

System prompt: You and I are scientists and researchers doing qualitative text analysis. The text is an interview of one surgical team member's perspectives on patient safety culture in a surgical context.

Prompt 1a: Use the file “Churruca 2021 dimensions PSC”on patient safety culture dimensions  and examine the file “LLM first interview translated for analysis“ from the perspective of patient safety culture

**AI:**

The file "Churruca 2021 dimensions PSC" defines several dimensions of patient safety culture. These include communication, feedback, teamwork, and error reporting, among others.

In the "LLM first interview translated for analysis", the interviewee discusses aspects of patient safety culture within their orthopedic section. They mention collaboration, openness, dialogue, and a non-hierarchical approach as being key to safe patient care. The interviewee also highlights the importance of learning from mistakes, having debriefings when necessary, and fostering a work environment where everyone feels comfortable discussing issues that may affect patient safety. These points align with several dimensions defined in the "Churruca 2021 dimensions PSC" file.

**Memo/ compared to Test 5a:**

The second paragraph is a short and adequate summary of the content from the text related to patient safety culture dimensions.

Prompt 1b: Examine the content in the text file “LLM first interview translated for analysis“ from the perspective of patient safety culture described in ” Churruca 2021 dimensions PSC”.

**AI:**

The text file "LLM first interview translated for analysis" presents a discussion about patient safety culture from the perspective of healthcare professionals within an orthopedic section. To analyze the content using the dimensions of patient safety culture (PSC) described in Churruca et al., 2021, we can examine how various aspects are addressed:

1. Involvement of patients in safety culture: The participants discuss the importance of openness and dialogue with the patients, acknowledging that they too share concerns about patient safety (as per the interviewer's statement). However, the direct engagement or collaboration with patients in improving safety is not discussed explicitly within this excerpt.
2. Interpersonal dynamics: The text mentions that hierarchy has been reduced, and there is more collaboration between healthcare professionals and a sense of safety in giving and receiving feedback. This implies a positive interpersonal dynamic where individuals are comfortable in sharing information to improve patient care.
3. Context of how safety is enacted in pressurized healthcare environments: The text mentions the importance of openness, debriefings, dialogue with management, and correction opportunities to ensure optimal patient safety when things go wrong. This suggests that there is a focus on addressing safety issues in a pressurized environment where mistakes may occur.

In conclusion, while the text does not explicitly address all dimensions mentioned by Churruca et al., it presents an engaging and collaborative work environment with a strong emphasis on patient safety. The participants discuss openness, dialogue, collaboration, debriefings, and continuous improvement – all crucial aspects of a positive patient safety culture. Further investigation may be needed to confirm if these practices are consistently implemented in the orthopedic section and their impact on patient outcomes.

**Memo/ compared to Test 5a:**

Point 1 is not related to the interview text. Point 2 can contribute to reflections, discussions and interview text. Point 2 and the concept “interpersonal dynamics” is based on content from the interview and can contribute to reflections, discussions and interpretations.

Prompt 2: Could you explore the interview text more in details to support my qualitative analysis of the interview text?

AI:

To provide a more detailed exploration of the interview text, I will analyze it using Churruca et al.'s dimensions of patient safety culture (2021), while considering additional aspects that may contribute to understanding the interviewee's perspectives on patient safety culture within their surgical context:

1. Involvement of patients in safety culture: The text mentions the importance of openness with patients, allowing them to express concerns and asking for their feedback. However, it is not explicitly stated whether patients are actively involved in decision-making related to improving patient safety.
2. Interpersonal dynamics: The interview highlights a positive interpersonal dynamic among team members. The surgical team member discusses the importance of collaboration with other healthcare professionals, such as nurses, physicians, and anesthesiologists, which contributes to a positive work environment that supports a strong patient safety culture.
3. Communication: Effective communication within the surgical team is emphasized as crucial for addressing issues promptly and ensuring optimal patient care. The text mentions openness in discussing mistakes and learning opportunities with peers, management, and patients, which promotes continuous improvement and further strengthens the patient safety culture.
4. Leadership: While not explicitly mentioned, the interview suggests that leadership plays a role in fostering a positive patient safety culture by promoting open dialogue, collaboration, and debriefings among team members. Additionally, the existence of clear channels for reporting incidents and concerns implies effective organizational structure and management support.
5. Continuous learning and improvement: The surgical team member mentions that they value learning opportunities from their mistakes and are committed to continuous professional development, which indicates a focus on improving patient safety through ongoing education and self-reflection.

In conclusion, the text highlights an engaging, collaborative work environment with a strong emphasis on openness, communication, and continuous improvement – all crucial aspects of a positive patient safety culture in surgical settings. By examining the interview from multiple perspectives, we can gain a deeper understanding of the interviewee's perspectives on patient safety culture and its implications for improving patient care within their surgical context.

**Memo/ compared to Test 5a:**

When asked to detail the response, LLM changes the third point to three new points that seem more far from the raw data and closer to Churruca`s paper.
