# Supplementary material for: Reflecting on LLM Support in Reflexive Thematic Analysis: An Exploratory Study
Source: Qual Health Res. 2025 Sep 8;36(2-3):191–205. doi: 10.1177/10497323251365211 (PMC12949038; doi:10.1177/10497323251365211)
Supplement: Supplemental Material - Reflecting on LLM Support in Reflexive Thematic Analysis: An Explorative Study [file sj-zip-1-qhr-10.1177_10497323251365211.zip › Supplemental_File_12_Test_7ab.docx]

Ollama Mistral Test 7

Translated text file and responses in English (language settings: English-GB).

Added the analysis method “reflexive thematic analysis” and the concept “culture” to the system prompt

Adding an extra document on patient safety culture (Churruca) in a single prompt.

Testing the connection between Donabedian and the text file/ the themes and subthemes in a separate chat is presented as 7b in this document.

System prompt: You and I are scientists and researchers doing qualitative text analysis using the method reflexive thematic analysis. We will analyze the file “LLM first interview.docx”. You are supposed to use this file and answer my prompts to support my reflexive thematic analysis. The text file is an individual interview of one surgical team member's perspectives on patient safety culture in a surgical context.

Prompt 1: Support phase 1 in the analysis: Summarize the text's main repetitive ideas and focus on significant patterns. The summary should be a maximum of 300 words. Do not add any content not present in the file.

**AI:** The text from the interview highlights several recurring themes regarding patient safety culture within a surgical context, as well as changes over time in this culture.

Firstly, there is a focus on collaboration between team members as being essential for safe patient care LLM first interview translated for analysis.docx. This collaborative approach has replaced a more hierarchical structure that was present in the past LLM first interview translated for analysis.docx. The interviewee recalls their earlier experiences as an operating room nurse, where everything had to be perfectly ready before the surgeons arrived and there wasn't much collaboration on making procedures simpler and safer LLM first interview translated for analysis.docx.

Secondly, feedback, both giving and receiving, is considered safe within this new collaborative culture, which was not always the case in the past LLM first interview translated for analysis.docx. This openness to feedback contributes to an environment where team members feel safe and more confident in their roles, particularly in high-risk settings like surgery LLM first interview translated for analysis.docx.

Lastly, there is a sense of internal humor used among team members that creates a comfortable and relaxed work atmosphere. This humor reinforces the collaborative culture and serves as a coping mechanism for dealing with the inherent risks associated with surgical procedures LLM first interview translated for analysis.docx.

In summary, the text demonstrates a shift from a more hierarchical and less collaborative approach to patient safety in the past, towards a current focus on collaboration, open feedback, and humor as key elements of a safe and effective patient safety culture within the surgical setting LLM first interview translated for analysis.docx.

**Memo/ compared to Test 5a and 5c:** As the summary in Test 5, this summary displays topics from the interview but excludes other essential topics. This summary does not present irrelevant details or created content. The summary focuses on collaboration, the changes in the hierarchy in the surgical context, the importance of feeling safe to give and receive feedback, and the internal humor that may facilitate a good work environment. The summary does not include important themes such as responses to errors and meetings for discussing risks and events. The summary may support the researcher and add new reflections, but can`t reduce the researcher`s time spent on familiarization.

**Prompt 2**: Support phase 2 in the analysis: A “code” is a phrase of 3-5 words representing a significant topic in the text. Suggest 20 “codes” from the file. Highlight semantic and latent meanings.

1. AI: Collaboration (Collaborative approach, Working together)
2. Hierarchy (Hierarchical structure, Commanding style)
3. Safety culture (Safety-focused environment)
4. Feedback (Giving feedback, Receiving feedback)
5. Experience (Past experiences, Learning from mistakes)
6. Support (Supportive colleagues, Supportive management)
7. Fun (Internal humor, Light atmosphere)
8. Patient (Patient safety, Safe patient care)
9. Readiness (Preparedness, Everything ready)
10. Attention (Standing at attention, Focused mindset)
11. Surgeon (Surgeons' presence, Collaboration with surgeons)
12. Simplicity (Making it simpler, Simple procedures)
13. Mistake (Mistakes made, Acknowledging mistakes)
14. Counting (Final count, Counting in and out)
15. Generational shift (Change over time, New generations)
16. Key (Collaboration as the key)
17. Coping mechanism (Using humor as a coping tool)
18. Locker room (Meeting place, Informal interactions)
19. Work atmosphere (Work environment, Work culture)
20. Paid for fun (Being paid to enjoy work)

**Memo/ compared to Test 5a and 5c:**

The “codes” are keywords from the text. The codes add more content to the summary/ familiarization (supportive colleagues/ management, meeting place), but the researcher need to examine whether they display the text file or not. In this response, no one of the codes are wrong or represent a high risk of bias. Some of the codes are without substance (locker room). The codes are not presented as “ideas”/ phrases, and despite the new content that is displayed, LLM does not contribute to the coding process. Asking for a more in-depth description after the summary could provide the same support.

**Prompt 3**: Support phase 3 in the analysis: The next step in this reflexive thematic analysis is to merge codes with similar content into 8-10 sub-themes. Create 8-10 sub-themes with appropriate names, identify which codes you have merged into each sub-theme, and each of the 8-10 sub-themes in 3 sentences.

**AI:**

1. Collaborative culture (Collaboration, Working together, Making it simpler)
   - The interviewee emphasizes the importance of collaboration between team members in ensuring safe patient care.
   - This shift from a hierarchical structure to a collaborative approach is considered crucial for improving safety culture within the surgical setting.
   - Collaboration also plays a role in making procedures simpler and safer, which can lead to increased efficiency and reduced risks.
2. Feedback (Giving feedback, Receiving feedback)
   - The openness to constructive feedback among team members is seen as essential for fostering an environment of learning and continuous improvement.
   - This new culture of giving and receiving feedback has replaced a more closed and hierarchical approach in the past.
   - Feedback is considered safe within this collaborative culture, contributing to increased confidence and better performance among team members.
3. Safety-focused environment (Patient safety, Safe patient care, Attention to details)
   - The interviewee recalls a more hierarchical approach to patient safety in the past compared to the current emphasis on collaboration and attention to detail.
   - A key element of this new safety culture is internal humor, which serves as a coping mechanism for dealing with the inherent risks associated with surgical procedures.
   - The focus on safety extends beyond just surgery, encompassing everything from preparation to recovery.
4. Support and mentorship (Supportive colleagues, Supportive management, Generational shift)
   - The interviewee mentions that support from both colleagues and management is essential for maintaining a positive work atmosphere.
   - This support is particularly important in high-risk settings like surgery, where team members must rely on each other to ensure patient safety.
   - A generational shift has brought about changes in the way collaboration, feedback, and mentorship are approached within the surgical team.
5. Emotional wellbeing (Stress management, Coping mechanisms, Fun)
   - The use of internal humor as a coping mechanism helps alleviate stress during high-pressure situations.
   - A relaxed work atmosphere contributes to emotional wellbeing and resilience among team members, which is crucial for maintaining focus and efficiency.
   - The interviewee notes that the fun aspect of their job is an important factor in their overall job satisfaction.
6. Learning from mistakes (Mistakes made, Acknowledging mistakes)
   - Mistakes are acknowledged as a natural part of any high-risk profession like surgery.
   - Constructive feedback and lessons learned from past mistakes help team members grow and improve over time.
   - The new culture of openness to feedback facilitates learning from mistakes, contributing to a safer environment for both patients and staff.
7. Role clarity (Role expectations, Role confidence)
   - Clarity in roles and responsibilities is essential for smooth collaboration within the surgical team.
   - A better understanding of each team member's role can lead to increased confidence and improved performance.
   - This role clarity is a key factor in promoting a collaborative culture where everyone feels valued and heard.
8. Team dynamics (Internal communication, Trust building, Relationships)
   - Effective internal communication within the surgical team is crucial for maintaining trust and fostering strong relationships among team members.
   - Building and maintaining these relationships can enhance collaboration and improve overall performance.
   - Internal humor serves as a tool for strengthening relationships and promoting a comfortable work atmosphere, which is conducive to better team dynamics.

**Memo/ compared to Test 5a and 5c:**

This response contributes to important nuances, concepts, and perspectives. LLM includes: Learning and continuous improvements, emotional well-being and resilience to foster and maintain focus and efficiency, learning from mistakes, role clarity/ responsibility, team dynamics, and relationships. The subthemes\ descriptions are overlapping, and this response immediately seems to be a solid support for the researcher, it requires that the researcher know the raw data well and examines whether LLM uses information about patient safety culture from sources other than the interview file. May the names of the subthemes be information about patient safety culture from other sources, and the descriptions be extracted from the interview file?

**Prompt 4:** Support phase 4 in the analysis: Organize sub-themes with similar content into 2 or 3 main themes with appropriate names and describe in-depth the essence of each main theme in 6 sentences.

**AI:**

1. Collaborative approach to patient safety (Collaboration, Support and mentorship, Learning from mistakes)
   - The interviewee emphasizes the importance of collaboration among team members in ensuring safe patient care within a surgical setting.
   - This collaborative culture is supported by constructive feedback, learning from past mistakes, and effective support systems for both colleagues and management.
   - A generational shift has brought about changes in the way collaboration, feedback, and mentorship are approached within the surgical team.
   - The new collaborative approach to patient safety has replaced a more hierarchical structure that was present in the past.
   - Collaboration plays a role in making procedures simpler and safer, which can lead to increased efficiency and reduced risks.
   - This shift towards collaboration also fosters a supportive work environment where team members feel comfortable communicating their concerns and suggestions.
2. Maintaining a safe and efficient surgical environment (Safety-focused environment, Emotional wellbeing, Role clarity)
   - The interviewee describes the importance of creating a safe and efficient surgical environment for both patients and staff.
   - Internal humor is used as a coping mechanism to alleviate stress during high-pressure situations while maintaining an overall relaxed work atmosphere.
   - Clarity in roles and responsibilities among team members contributes to increased confidence and improved performance, which in turn enhances the safety culture.
   - A supportive work environment helps employees manage stress, cope with difficult situations, and maintain emotional wellbeing.
   - The focus on safety extends beyond just surgery, encompassing everything from preparation to recovery.
   - Effective internal communication within the surgical team is crucial for maintaining trust, fostering strong relationships, and promoting a positive work atmosphere conducive to better team dynamics.

**Memo/ compared to Test 6:**

This response does not contribute with any new nuances, concepts or perspectives. The themes are not generated in an appropriate way.

**~~Prompt 5:~~** ~~Support phase 5 in the analysis:~~ ~~Refine the sub-theme names and theme names to a more latent, nuanced and interpretative level: (Paste sub-themes and themes.) OUT~~

**~~Prompt 6:~~** ~~Find and extract specific and direct quotes from the text file to illustrate each sub-theme from phase 4. Do not change the sentences you pick from the original text file.~~ High risk of bias.

**~~Prompt 6:~~** ~~Do any identified themes or sub-themes relate to or interact with each other? Describe any connections you observe: (Paste themes and sub-themes) OUT~~

**~~Prompt 7:~~** ~~Critically evaluate this conversation and the file from the perspective of Donabedian’s theory of quality in healthcare services.~~ Separate test.

**Prompt 5a:**

Examine the “LLM first interview translated for analysis“file from the theoretical perspective of patient safety culture presented in the added file “Churruca 2021 dimensions PSC“.

**AI:**

1. Support phase 1 in the analysis: Identify key phrases or "codes" that capture significant aspects related to the concept of Patient Safety Culture (PSC) in the text file.

- Collaborative approach to patient safety
- Safety-focused environment
- Emotional wellbeing and stress management
- Role clarity
- Learning from mistakes
- Clear communication and trust building
- Internal humor as a coping mechanism

1. Support phase 2 in the analysis: Merge codes with similar content into sub-themes, ensuring each sub-theme aligns with one or more of the dimensions presented in the theoretical perspective of PSC (Churruca 2021).

- Sub-theme 1: Collaborative culture for patient safety
  - Collaboration and teamwork
  - Support and mentorship among colleagues and management
  - Learning from mistakes
- Sub-theme 2: Safety-focused environment
  - Patient safety and safe patient care
  - Attention to details
  - Internal humor as a coping mechanism
- Sub-theme 3: Stress management, emotional wellbeing, and resilience
  - Coping mechanisms for stress
  - Emotional wellbeing and support systems
  - Building relationships and trust among team members
- Sub-theme 4: Clear roles and responsibilities
  - Role clarity and expectations
  - Role confidence
  - Team dynamics and communication

1. Support phase 3 in the analysis: Synthesize sub-themes into main themes that align with the dimensions presented in the theoretical perspective of PSC (Churruca 2021).

- Main theme 1: Collaborative approach to patient safety culture
  - Encourages collaboration and teamwork among staff
  - Fosters learning from mistakes
  - Supports open communication, trust building, and relationship-building
- Main theme 2: Safety-focused environment
  - Promotes a strong focus on patient safety
  - Utilizes internal humor as a coping mechanism to maintain a relaxed work atmosphere
  - Ensures attention to details for safe patient care

1. Support phase 4 in the analysis: Reflect on the findings and consider potential implications for practice, research, or policy related to Patient Safety Culture (PSC).

- Implication for Practice: Encourage collaborative approaches to patient safety among surgical teams and emphasize the importance of learning from mistakes. Foster open communication, trust building, and relationship-building within surgical teams. Implement stress management strategies and support systems to promote emotional wellbeing and resilience among staff.
- Implication for Research: Further investigate how collaborative approaches to patient safety culture contribute to improved outcomes in surgical settings. Examine the impact of internal humor as a coping mechanism on employee stress, emotional wellbeing, and overall team dynamics. Investigate the role of clear roles and responsibilities in enhancing team performance and promoting a safety-focused environment.
- Implication for Policy: Develop policies that support collaborative approaches to patient safety culture and encourage open communication, trust building, and relationship-building within surgical teams. Implement stress management strategies and support systems to promote emotional wellbeing and resilience among staff. Establish clear roles and responsibilities for team members in order to improve performance and maintain a strong focus on patient safety.

**Memo/ compared to Test 6:**

LLM presents the response as the analysis process, but the referred phases are wrong. It seems like the LLM has used Churruca in the previous responses based on the attached files in previous chats. The response on implications is based on the prompts in previous chats. High risk of bias in this response, and no contributions.

**Prompt 5b:**

Examine the themes you generated (“Collaborative approach to patient safety” and “Maintaining a safe and efficient surgical environment”) and subthemes (“Collaborative culture”, “Feedback”, “Safety-focused environment“, “Support and mentorship“, Emotional wellbeing”, “Learning from mistakes“, “Role clarity”) from the theoretical perspective of patient safety culture presented in the added file “Churruca 2021 dimensions PSC“

**AI:**

1. Collaborative approach to patient safety
   - The collaborative approach to patient safety aligns with the interdependent dimension of patient safety culture as described by Churruca (2021). In this interview, the surgical team member emphasizes the importance of collaboration and support among team members in ensuring safe patient care. This is evident in subthemes such as "Collaborative culture", "Support and mentorship", and "Learning from mistakes".
   - The interdependent dimension of patient safety culture also encompasses a mutual trust, respect, and accountability among team members. In this interview, the surgical team member notes that effective internal communication is crucial for maintaining trust and fostering strong relationships within the surgical team. This supports Churruca's (2021) assertion that interdependent dimensions help create a culture where everyone feels responsible for patient safety.
   - The focus on collaboration in this interview also aligns with the communication dimension of patient safety culture, as described by Churruca (2021). Effective communication and feedback are essential to ensure all team members understand their roles and responsibilities, which can lead to increased confidence and improved performance. This is reflected in subthemes such as "Role clarity" and "Feedback".
2. Maintaining a safe and efficient surgical environment
   - The focus on maintaining a safe and efficient surgical environment aligns with the organizational learning dimension of patient safety culture, as described by Churruca (2021). In this interview, the surgical team member notes the importance of creating a supportive work environment that promotes emotional wellbeing and effective coping mechanisms. This is evident in subthemes such as "Emotional wellbeing" and "Learning from mistakes".
   - The focus on maintaining a safe and efficient surgical environment also aligns with the teamwork within multidisciplinary teams dimension of patient safety culture, as described by Churruca (2021). In this interview, the surgical team member highlights the importance of role clarity and communication among team members. This is evident in subthemes such as "Role clarity" and "Communication".
   - The focus on maintaining a safe and efficient surgical environment also aligns with the safety awareness and psychological empowerment dimensions of patient safety culture, as described by Churruca (2021). In this interview, the surgical team member emphasizes the importance of a safety-focused environment where everyone feels responsible for ensuring patient safety. This is evident in subthemes such as "Safety-focused environment" and "Learning from mistakes".

**Memo/ compared to Test 6:**

The response does not provide any new perspectives, and since the researcher should know their theory, this response will have minimal benefits. The descriptions are overlapping. At best, this response can be used as basic for discussions and reflections, but the research panel should be aware of the risk of bias.

**Prompt 6:** Based on this conversation and the file “LLM first interview translated for analysis”: Summarize the primary insights and clinical implications for healthcare services.

**AI:**

1. Primary insights
   - The interviewed surgical team member emphasizes the importance of collaboration and support among team members in ensuring safe patient care within a surgical setting. This collaborative approach aligns with the interdependent dimension of patient safety culture, as described by Churruca (2021).
   - The focus on maintaining a supportive work environment that promotes emotional wellbeing and effective coping mechanisms supports the organizational learning dimension of patient safety culture.
   - Effective communication and feedback are essential to ensure all team members understand their roles and responsibilities, which aligns with the communication dimension of patient safety culture.
   - The focus on a safety-focused environment where everyone feels responsible for ensuring patient safety supports the safety awareness and psychological empowerment dimensions of patient safety culture.
2. Clinical implications for healthcare services
   - To improve patient safety culture in surgical settings, healthcare providers should prioritize collaboration and support among team members to ensure safe patient care. This can be achieved by fostering a culture where everyone feels responsible for patient safety and open communication is encouraged.
   - Healthcare organizations can promote emotional wellbeing and effective coping mechanisms among employees by creating supportive work environments that address stressors, provide resources for mental health support, and encourage feedback and learning from mistakes.
   - Effective communication and role clarity are crucial to ensure all team members understand their roles and responsibilities in ensuring patient safety. Regular team meetings, clear job descriptions, and opportunities for ongoing education can help improve communication and role clarity.
   - To promote a safety-focused environment, healthcare organizations should emphasize the importance of psychological empowerment, encouraging employees to take ownership of their roles in ensuring patient safety. This can be achieved by providing resources and support for employees to learn about patient safety, offering opportunities for feedback and continuous improvement, and recognizing employee contributions to patient safety efforts.

**Memo/ compared to Test 6:**

No new contributions. As observed in previous conversations, the response refers to the attached file in the previous prompt (only once).

**Test 7b Donabedian- separate chat**

Added the file “LLM first interview translated for analysis” and the file “Donabedian 1966”

**System prompt:** You and I are scientists and researchers doing qualitative text analysis. The text file is an individual interview of one surgical team member's perspectives on patient safety culture in a surgical context.

**Prompt:** Use the file on Donabedian’s theory of quality in healthcare services and examine the file “LLM first interview translated for analysis“ in Donabedian`s framework.

**AI:**

According to Donabedian's theory, quality in healthcare services can be assessed at both the individual patient-physician level and at the population level Donabedian 1966.pdf. In the "LLM first interview translated for analysis," the focus seems to be more on the individual patient (ASA 3, ASA 4) and their care within the operating room, rather than on the quality of medical care received by a larger population group or the efficiency of providing and delivering services Donabedian 1966.pdf.

However, the interviewee does touch upon factors that could be related to the broader Donabedian framework, such as accessibility (implication of sick patients and working more efficiently) and utilization (implication of a different pattern of use affecting quality) Donabedian 1966.pdf.

It is worth noting that the interviewee also mentions management's focus on work efficiency, which could be seen as an effort to improve the system and services provided at the population level, even though the main concern appears to be individual patients within their closed operating room world [LLM first interview translated for analysis].

Overall, while there is some alignment with Donabedian's framework in the "LLM first interview translated for analysis," the focus remains more on the individual patient-physician relationship. The broader aspects of quality concerning the distribution, accessibility, and use of medical services, as well as socioeconomic variables, are not extensively discussed

**Memo/ compared to Test 6:**

LLM does not reflect on the text in the framework of Donabedian. No contributions. Interestingly, this test provides less support than 5b, which is similar.

**Prompt:** Examine the themes (“Collaborative approach to patient safety” and “Maintaining a safe and efficient surgical environment”) and subthemes (“Collaborative culture”, “Feedback”, “Safety-focused environment“, “Support and mentorship“, Emotional wellbeing”, “Learning from mistakes“, “Role clarity”) from the file "LLM first interview translated for analysis" in the perspective of Donabedian’s theory of quality in healthcare services.

**AI:**

In Donabedian's theory of quality in healthcare services, the focus is on improving the quality of medical care received by population groups, not just individuals (Donabedian 1966). The themes and subthemes discussed in "LLM first interview translated for analysis" can be analyzed from this perspective as follows:

- Collaborative approach to patient safety: This theme aligns with Donabedian's emphasis on collaboration and population-level studies. In a collaborative approach, healthcare providers work together to ensure safe and efficient care for all patients, rather than just focusing on individual patients.
- Maintaining a safe and efficient surgical environment: This theme relates to the distribution and accessibility of medical services (Donabedian 1966). A safe and efficient surgical environment is necessary for ensuring high-quality care for all patients.
- Collaborative culture: In Donabedian's theory, collaboration between healthcare providers is essential for improving quality. The collaborative culture discussed in the interview highlights the importance of teamwork and cooperation among healthcare professionals.
- Feedback: Providing feedback to healthcare providers is important for identifying areas for improvement and for ensuring that best practices are being followed. This aligns with Donabedian's emphasis on studying outcomes and using this information to make improvements.
- Safety-focused environment: A safety-focused environment is necessary for ensuring high-quality care. In Donabedian's theory, the quality of medical care received by a population group can change simply through a different pattern of use (Donabedian 1966).
- Support and mentorship: Providing support and mentorship to new healthcare providers is important for ensuring that they are able to provide high-quality care. This aligns with Donabedian's focus on organizational changes and the importance of introducing best practices (Donabedian 1966).
- Emotional wellbeing: The emotional wellbeing of healthcare providers is important for ensuring that they are able to provide high-quality care. In Donabedian's theory, quality is influenced by the issues and values of the medical care system (Donabedian 1966).
- Learning from mistakes: In Donabedian's theory, studying outcomes and learning from mistakes is important for identifying areas for improvement and for making improvements to the quality of care. This aligns with the emphasis on learning from mistakes discussed in the interview.
- Role clarity: Role clarity is important for ensuring that healthcare providers are able to provide high-quality care. In Donabedian's theory, role clarity is necessary for studying outcomes and for making improvements to the quality of care (Donabedian 1966).

In summary, the themes and subthemes discussed in "LLM first interview translated for analysis" align with Donabedian's theory of quality in healthcare services. The emphasis on collaboration, population-level studies, learning from mistakes, and role clarity are all key components of Donabedian's theory.

**Memo/ compared to Test 6:**

The three main categories in the framework of Donabedian are not mentioned. This requires that the researcher knows the raw data and the attached files well (again).
