# Supplementary material for: Reflecting on LLM Support in Reflexive Thematic Analysis: An Exploratory Study
Source: Qual Health Res. 2025 Sep 8;36(2-3):191–205. doi: 10.1177/10497323251365211 (PMC12949038; doi:10.1177/10497323251365211)
Supplement: Supplemental Material - Reflecting on LLM Support in Reflexive Thematic Analysis: An Explorative Study [file sj-zip-1-qhr-10.1177_10497323251365211.zip › Supplemental_File_13_Consent_Form.docx]

As an addition to the project

‘Patient Safety Culture and Adverse Events’

we ask for your consent to use the deidentified interview texts for re-analysis using artificial intelligence

This is a question about whether we can use your de-identified interview data from the project 'Patient Safety Culture and Adverse Events' and conduct a re-analysis using artificial intelligence (AI). In this document, we provide information about the study's purposes and what further use of these data will entail for you.

**Purpose**The purpose of the study is to acquire new knowledge about how AI tools can be used in the initial phases of qualitative analysis and whether these tools can make these phases of the analysis process more efficient. Secondly, we aim to test further analysis and results with AI and to evaluate and compare the results with the original thematic analysis that has been performed and published.

The study is part of the research project 'Patient Safety Culture and Adverse Events,' a PhD project.

(Anon) is responsible for the project.

**Why are you being asked to participate?** Since you participated in the original study, you are being asked if we can use the de-identified data from your interview.

**What does participation entail for you?** Participation means that the de-identified transcribed text from your interview will be included in a new analysis. All you need to do is to provide consent electronically via the link to the online form included in the email where this consent form is attached.

**Participation is voluntary.**It is voluntary to approve the further use of your interview data in a re-analysis. If you choose to consent, you can withdraw your consent anytime without providing any reason. Your de-identified data will be removed from the re-analysis, provided the study has not been published. You will have no negative consequences if you choose not to participate or later decide to withdraw.

**Your privacy – how we store and use your information:**

We will only use the de-identified interview data for the purposes outlined in this document. We will handle the information confidentially and in accordance with data protection regulations.

- Only the PhD candidate and the main supervisor have access to the information collected from the original study.
- Information collected from the original study: Your name has been replaced with a code that is stored along with the characteristics obtained during the interview. The form with the characteristics and consent forms with contact information is stored securely, and audio recordings are encrypted and stored in (Anon).
- The transcribed text material from the original study is now stored in (Anon) Service for Sensitive Data.
- We will use Sikt AI chat, or an equivalent AI tool, as the instrument for the re-analysis. The instrument used will ensure data privacy in accordance with GDPR.
- The rest of the project group will only have access to de-identified data. De-identified means that all directly and indirectly identifiable information has been removed or reformulated.
- You will not be identifiable in the publication, and the content of any future article will primarily be methodological.

**What happens to your data when the research project ends?**The data will be deleted when the project ends/the thesis is approved, planned for 31.05.2028. Consent forms, notes, coded forms with personal data, and transcribed material will be disposed of following regulations, and audio files will be deleted at the project's conclusion.

**What gives us the right to process your personal data?**We process your data based on your consent. On behalf of (Anon), The Norwegian Agency for Shared Services in Education and Research, Sikt – the Service Provider for the Knowledge Sector has assessed that the processing of personal data in this project follows data protection regulations.

**Your Rights**

As long as you can be identified in the data material, you have the right to:

- access the information we process about you and receive a copy of the data
- have incorrect or misleading information about you corrected
- have your personal data deleted
- file a complaint with the Data Protection Authority regarding the processing of your personal data

If you have any questions about the study or wish to exercise your rights, please contact: (Anon)

Our Data Protection Officer: (Anon)

If you have any questions regarding the assessment made by Sikt's data protection services, you can contact them via email at: (Anon)

Best regard,

(Anon) (Anon)

Main supervisor/ researcher PhD Candidate

-------------------------------------------------------------------------------------------------------------------------

**Consent Form**

I have received and understood the information about the project “LLM-support in Reflexive Thematic Analysis” and have had the opportunity to ask questions.

I consent to:

□ The de-identified text material from my interview is being used to re-analyze with Sikt AI Chat.

Use the link in the separate e-mail

----------------------------------------------------------------------------------------------------------------

Signature
