# Supplementary material for: Reflecting on LLM Support in Reflexive Thematic Analysis: An Exploratory Study
Source: Qual Health Res. 2025 Sep 8;36(2-3):191–205. doi: 10.1177/10497323251365211 (PMC12949038; doi:10.1177/10497323251365211)
Supplement: Supplemental Material - Reflecting on LLM Support in Reflexive Thematic Analysis: An Explorative Study [file sj-zip-1-qhr-10.1177_10497323251365211.zip › Supplemental_File_3_Test_2_Norwegian.docx]

Ollama Mistral Test 2- answers in Norwegian

System prompt: You and I are scientists and researchers doing qualitative text analysis. Use the file “LLM first interview.docx” and answer my prompts to support my qualitative analysis. The text is an interview of a surgical team member's perspectives on patient safety in a surgical context. Generate all your answers in Norwegian.

Prompt 1: First step: Summarize the main ideas of the text. Focus on significant patterns or repetitive ideas. The summary should be a maximum of 150 words. Do not add any content that is not present in the file.

**AI:** I summarize the main ideas of the provided interview as follows: The interviewee emphasizes that patient safety is central to their work, which they perceive as a shared responsibility among team members [source_id LLM first interview.docx]. They express that the team's sense of security and well-being is essential for achieving a focus on patient care and safety [source_id LLM first interview.docx]. Additionally, the interviewee mentions that they understand the need to prioritize salaries but prefers to discuss other matters while at work, focusing more on patient care instead [source_id LLM first interview.docx]. Lastly, the interviewee indicates a deep dedication to their work in the operating division and finds joy in contributing to patient care, as they view it as a privilege to be part of a team that can make a significant impact on patients' lives [source_id LLM first interview.docx].

**Memo/ compared to Test 1:**

LLM generated responses in English. The summary is written more coherently and highlights other topics than Test 1: Shared responsibility, well-being, focus on patient safety, deep dedication, joy, and privilege to be a part of the team. This summary lacks the topics workload, resources, dialogue, and collaboration. Conclusion: This prompt supports reflexivity more than Test 1. Suggestion: Increased number of words may add more ideas from this information-rich text.

**Prompt 2a**: Second step: A “code” is a phrase of 2-5 words representing a significant topic in the text. Suggest 20 “codes” from the file. Highlight semantic meaning.

1. AI: *Pasientsikkerhet (patientsafety)* - Påstanden at pasientesikkerheten er central i det arbeide som intervieween gjør.
2. *Dialog (dialogue)* - Intervjuet understrekker dialogens veldig viktige rolle i sikre å oppnå pasientsikkerhet.
3. *Team (teamwork)* - Intervieuende mener at at det er utrolig viktig å arbeide i team for å kunne gjøre en god jobb og sikre pasientesikkerheten [source_id LLM first interview.docx].
4. *Pasienter har lite vondt (patients have little pain)* - Intervieuende mener at det er et eksempel på hvorfor det er viktig å sette pasientene i sideleie for å de skal ha mindre vondt [source_id LLM first interview.docx].
5. *Sideleie (prone position)* - Intervieuende mener at at det er en metode som kan reduksjon av smårørelse og pasientens vondt i sittende stilling [source_id LLM first interview.docx].
6. *Anestesi (anesthesia)* - Interviuet omhandler spesielt anestesiiske aspekter av pasientsikkerheten i en kirurgisk kontekst [source_id LLM first interview.docx].
7. *Kveld og dagtid (daytime and evening)* - Intervieuende mener at det legger godt merke til å seksjonert på anestesiiske aspekter av pasientsikkerheten både under dagtid og kveld [source_id LLM first interview.docx].
8. *Salarier (salaries)* - Intervieuende mener at det legger godt merke til hvorfor det er viktig å prioritere salarier, men fokuserer på andre temaer under arbeidstiden [source_id LLM first interview.docx].
9. *Anestesisykepleiere og doktorer (anesthesia nurses and doctors)* - Interviuet omhandler det faktum at anestesiiske aspekter av pasientsikkerheten er seksjonert mellom anestesisykepleiere og doktorer [source_id LLM first interview.docx].
10. *Sikre (ensure)* - Interviuet understrekker hvor mye det er viktig å sikre pasientsikkerheten i en kirurgisk kontekst [source_id LLM first interview.docx].
11. *Kan du se vem som er på jobb (can you see who is on duty)* - Intervieuende understrekker hva viktig det er å se hvem som er på jobb for å kunne hjelpe sannsynligvis mer effektivt [source_id LLM first interview.docx].
12. *På dagtid (during the day)* - Intervieuende understrekker hva viktig det er å prioritere pasientsikkerheten under dagtid, da anestesiiske aspekter av pasientsikkerheten er seksjonert [source_id LLM first interview.docx].
13. *Anstende (dedicate)* - Intervieuende uttrykker en dyp dedikasjon til arbeide i operasjonssaks som inneholder en rolle i pasientsikkerheten [source_id LLM first interview.docx].
14. *En privilegium (a privilege)* - Intervieuende ser det som et privilegium å kunne bidra til forbedring av pasienters liv i operasjonssaks [source_id LLM first interview.docx].
15. *På dagtid og kveld (daytime and evening)* - Interviuet understrekker hva viktig det er å sikre en kontinuitet i arbeidsforholdet både under dagtid og kveld [source_id LLM first interview.docx].
16. *Anestesiiske aspekter (anesthesia aspects)* - Interviuet omhandler hvordan anestesiiske aspekter kan sikre pasientsikkerheten i en kirurgisk kontekst [source_id LLM first interview.docx].
17. *Hjelp (help)* - Intervieuende understrekker hva viktig det er å lese litt på veien for å kunne hjelpe pasienten mer effektivt [source_id LLM first interview.docx].
18. *Effektiv arbeidsforhet (effective work environment)* - Intervieuende mener at det legger godt merke til hva viktig et effektivt arbeidsforhet er for å kunne sikre pasientsikkerheten [source_id LLM first interview.docx].
19. *Arbeider i team (working in a team)* - Interviuet understrekker hva viktig det er å arbeide i en hold som kan bidra til pasientsikkerheten i operasjonssaks [source_id LLM first interview.docx].
20. *Pasientens vondt (patient's pain)* - Interviuet omhandler hvordan pasienten kan reduksjon av smårørelse og vondt i sittende stilling ved å sette dem i sideleie [source_id LLM first interview.docx].

**Memo/ compared to Test 1:** Most of the codes are keywords. Many of them overlap exactly. The codes are translated into English and described in poor Norwegian. These descriptions say something about the content but cannot support the researcher in creating codes. The keywords are semantic, descriptive words.

**Prompt 2b**: Second step: A “code” is a phrase of 2-5 words representing a significant topic in the text. Suggest 20 “codes” from the file. Highlight latent meaning.

**AI:** Vi er to forskere og tekstanalytikere som jobber med å analyse den kvalitative teksten fra filen "LLM first interview.docx". Teksten består av et intervju med en operasjonsmedlem som uttrykker sin holdning til pasientsikkerhet i en kirurgisk kontekst. Vi skal her avhenge 20 koder fra teksten, og høye opp de latente betydningene.

1. *Pasientesikkerhet (patientsafety)* - Påstanden at pasientesikkerheten er central i det arbeide som intervieween gjør.
2. *Dialog (dialogue)* - Intervjuet understrekker dialogens viktig rolle for å oppnå pasientsikkerhet.
3. *Team (teamwork)* - Intervieuende mener at det er viktig å arbeide i team for å sikre pasientesikkerhet.
4. *Arbeider i team (working in a team)* - Understrekker hva viktig det er å arbeide sammen for å sikre pasientsikkerhet.
5. *Effektivt arbeidsforhet (effective work environment)* - Intervieuende understrekker hva viktig det er å ha en effektivt arbeidsforhet for å sikre pasientesikkerhet.
6. *Arbeider på dagtid og kveld (working daytime and evening)* - Understrekker hva viktig det er å arbeide både under dagtid og kveld for å sikre pasientesikkerhet.
7. *Hjelp pasienten (help the patient)* - Intervieuende understrekker hva viktig det er å lese litt på veien for å kunne hjelpe pasienten effektivt.
8. *Anstende seg til operasjonssaksen (dedicate oneself to surgery)* - Intervieuende uttrykker en dyp dedikasjon til arbeide i operasjonssaks.
9. *Påverke pasientens vondt (affect patient's pain)* - Understrekker hva viktig det er å se på paverkingen av pasientens vondt for å sikre pasientesikkerhet.
10. *Sette pasienten i sideleie (put the patient in the lateral position)* - Understrekker en metode for å reduksjon av pasientens smårørelse og vondt.
11. *Se hvem som er på duty (see who is on duty)* - Understrekker hva viktig det er å se hvem som er på jobb for å kunne hjelpe pasienten effektivt.
12. *Kontinuitet i arbeidsforholdet (continuity in work environment)* - Understrekker hva viktig kontinuitet i arbeidsforholdet er for å sikre pasientesikkerhet.
13. *Sannsynligvis mer effektivt (more likely more effective)* - Understrekker at det kan være sannsynligvis mer effektivt å lese litt på veien først.
14. *Reduksjon av smårørelse og vondt (reduction of pain and discomfort)* - Understrekker en mål for å sikre pasientesikkerhet.
15. *Hva er pasientens hovedproblem (what is the patient's main problem)* - Understrekker hvordan man kan se på pasienten for å kunne hjelpe dem effektivt.
16. *På dagtid og kveld (daytime and evening)* - Understrekker hva viktig det er å sikre en kontinuitet i arbeidsforholdet både under dagtid og kveld.
17. *Sekundær problem (secondary problems)* - Understrekker hvordan man kan se på pasienten for å kunne identifisere sekundære problemer.
18. *Anstende seg til arbeidet (dedicate oneself to work)* - Understrekker en del av intervieuendenes holdning til arbeide i operasjonssaks.
19. *En privilegium (a privilege)* - Intervieuende ser det som et privilegium å kunne bidra til forbedring av pasienters liv i operasjonssaks.
20. *Se hva som er på veien (see what is on the way)* - Understrekker hva viktig det er å lese litt på veien for å kunne hjelpe pasienten effektivt.

**Memo/ compared to Test 1:** Generally, the responses were the same as the LLM provided when we asked for semantic codes. LLM added continuity, which is relevant and important. The word continuity is not used directly in the interview text. This prompt may be eliminated, and “latent” may be included in 2b.

**Prompt 3**: The third step in this qualitative analysis is to merge codes with similar content into eight to ten sub-themes. Create eight to ten sub-themes and give them appropriate and nuanced names. Identify which codes you have merged into each sub-theme.

**AI:** Trettede trinn i denne kvalitativ analyse er å samle koder med liknende innhold til åttetre og ti hovedtemaer. Opprette åttetre til ti hovedtemaer og gi dem passende og nuansete navn. Identifisere koder som er samlet i hver hovedtema.

1. *Pasientesikkerhet (patientsafety)* : Kodene "Pasientesikkerhet", "Effektivt arbeidsforhet", "Kontinuitet i arbeidsforholdet" og "Sekundær problem" har samlet seg til hovedtemaet "Pasientesikkerhet".
2. *Dialog (dialogue)* : Kodene "Dialog" og "Anstende seg til operasjonssaksen" har samlet seg til hovedtemaet "Dialog og dedikasjon".
3. *Arbeider i team (working in a team)* : Koderne "Arbeider i team", "Arbeider på dagtid og kveld" og "Anstende seg til arbeidet" har samlet seg til hovedtemaet "Samarbeid og dedikasjon".
4. *Hjelp pasienten (help the patient)* : Kodene "Hjelp pasienten", "Reduksjon av smårørelse og vondt" og "Sannsynligvis mer effektivt" har samlet seg til hovedtemaet "Hjelpepasient".
5. *Sette pasienten i sideleie (put the patient in the lateral position)* : Koderne "Sette pasienten i sideleie" og "Påverke pasientens vondt" har samlet seg til hovedtemaet "Reduksjon av smårørelse og vondt".
6. *Se hvem som er på duty (see who is on duty)* : Koderne "Se hvem som er på duty", "Hva er pasientens hovedproblem" og "En privilegium" har samlet seg til hovedtemaet "Identifisering av problem".
7. *Leste litt på veien (read a little on the way)* : Koderne "Se hva som er på veien", "Hvem som er på duty" og "Leste litt på veien" har samlet seg til hovedtemaet "Forberedelse".
8. *Reduksjon av smårørelse og vondt (reduction of pain and discomfort)* : Kodene "Reduksjon av smårørelse og vondt", "Se hvem som er på duty" og "Påverke pasientens vondt" har samlet seg til hovedtemaet "Reduksjon av smårørelse og vondt".

**Memo/ compared to Test 1:** Some of the codes are merged into more than one sub-theme. The introduction of the response indicates that the given step number and the number of sub-themes in the prompt need to be clarified. This step does not contribute to new nuances, concepts, or perspectives.

**Prompt 4:** Use content from the file to write a description of each of the eight to ten sub-themes.

**AI:** Vi er to forskere og tekstanalytikere som jobber med å analyse den kvalitative teksten fra filen "LLM first interview.docx". Teksten består av et intervju med en operasjonsmedlem som deler sine synspunkter på patient safety i en surgical context. Vi oppretter åttetre til ti hovedtemaer og beskriver de nedenfor.

1. *Pasientesikkerhet (patient safety)* : Denne temaet omhandler operasjonsteknisk sikkerhet for patienten, inkludert god planlegging, effektivt arbeidsforhet, kontinuitet i arbeidsforholdet og reduksjon av risiko for sekundære problemer.
2. *Dialog (dialogue)* : Denne temaet omhandler den dialog som oppstår mellom operasjonsmedlemmene for å diskutere patientens tilstand, behandlingsveier og andre aspekter av operasjonen for å sikre en optimal resultat.
3. *Arbeider i team (working in a team)* : Dette temaet omhandler samarbeid og kommunikasjon mellom operasjonsmedlemmene for å sikre at de arbeider effektivt sammen til å nå fremgangsmåtene og oppnå det ønskede resultatet.
4. *Hjelp pasienten (help the patient)* : Dette temaet omhandler hvordan operasjonsmedlemmene forsørger seg for å hjelpe patienten og sikre at de oppnår det ønskede resultatet, inkludert reduksjon av smårørelse og vondt.
5. *Reduksjon av smårørelse og vondt (reduction of pain and discomfort)* : Dette temaet omhandler reduksjon av smårørelse og vondt i samband med operasjoner, inkludert hvordan dette kan oppnås ved å bruke effektive metoder.
6. *Identifisering av problem (identifying problems)* : Dette temaet omhandler hvordan operasjonsmedlemmene oppdagler og hanterer problemer for å sikre at de kan gjerne løse dem på en effektiv måte før de blir vanvittigheter.
7. *Forberedelse (preparation)* : Dette temaet omhandler hvordan operasjonsmedlemmene forbereder seg for å gjere beste mulig forberedelse av patienten og oppnå det ønskede resultatet, inkludert lese litt på veien.
8. *En privilegium (a privilege)* : Dette temaet omhandler operasjonsmedlemmens følelser av at det er et privilegium å kunne bidra til forbedring av patienters liv i en surgical context.

**Memo/ compared to Test 1:** LLM changes some of the subthemes’ names when we ask for descriptions. Some of the description is overlapped. One word is highlighted compared to the codes: Preparations. A new perspective of how the members identify risk and adjust their performance before injuries emerge (described in less academic phrasing/ poor Norwegian, but presents the same meaning). Merge prompts 3 and 4?

**Prompt 5:** Organize sub-themes with similar content into two to three main themes and give the themes appropriate and nuanced names.

**AI:** Vi er to forskere og tekstanalytikere som jobber med å analyse den kvalitative teksten fra filen "LLM first interview.docx". Teksten består av et intervju med en operasjonsmedlem som deler sine synspunkter på patient safety i en surgical context. Vi samler koder med liknende innhold til to til tre hovedtemaer og gir dem passende og nuansete navn.

1. *Patient sikkerhet (patient safety)* : Denne temaet omhandler operasjonsteknisk sikkerhet for patienten, inkludert god planlegging, effektivt arbeidsforhet, kontinuitet i arbeidsforholdet og reduksjon av risiko for sekundære problemer. Kodene som er samlet til denne hovedtemaet er "Pasientesikkerhet", "Effektivt arbeidsforhet", "Kontinuitet i arbeidsforholdet" og "Sekundær problem".
2. *Dialog (dialogue)* : Denne temaet omhandler den dialog som oppstår mellom operasjonsmedlemmene for å diskutere patientens tilstand, behandlingsveier og andre aspekter av operasjonen for å sikre en optimal resultat. Kodene som er samlet til denne hovedtemaet er "Dialog" og "Anstende seg til operasjonssaksen".
3. *Arbeide i team (working in a team)* : Dette temaet omhandler samarbeid og kommunikasjon mellom operasjonsmedlemmene for å sikre at de arbeider effektivt sammen til å nå fremgangsmåtene og oppnå det ønskede resultatet. Kodene som er samlet til denne hovedtemaet er "Arbeider i team", "Arbeider på dagtid og kveld" og "Anstende seg til operasjonssaksen".

**Memo/ compared to Test 1:** The introduction of the response presents that LLM will merge codes into themes. These “codes” they present are neither the 20 codes nor the eight sub-themes. The themes are topic summaries that represent the content of the dataset. However, a human familiarization should recover the same topics/ keywords.

**Prompt 6:** Use content from the interview to write a description of each theme. Provide a description that captures their essence in one to two sentences.

**AI:** Vi er to forskere og tekstanalytikere som analyserer den kvalitative teksten fra filen "LLM first interview.docx". Teksten er et intervju med en medlem av en operasjonshold som deler sine synspunkter på patient safety i en surgical context. Vi skal bruke innholds fra intervjuet for å skrive en beskrivelse av hver tema, som fanger essensen i én-tveit sentanse.

1. *Sikkerhet gjennom planlegging (Sikkerhet gjennom planlegging)* : Operasjonsteknisk sikkerhet er nøkkelen til en succesfuld operasjon, og det står klart at god planlegging er viktig for å sikre en optimal resultat.
2. *Dialog mellom medlemmene av holdet (Dialog mellom medlemmene av holdet)* : Dialogen mellem medlemmene av operasjonsholdet er viktig for å nå fremgangsmåtene og oppnå det ønskede resultatet. Det står klart at hva som skjer på scenen må oppfattes som et helhetsperspektiv og gjenopplastes i dialogen medholdene for å sikre at de kan løse eventuelle problemer på en effektiv måte før de blir vanvittigheter.
3. *Arbeide i team (Arbeide i team)* : For å nå fremgangsmåtene og oppnå det ønskede resultatet er det viktig forhold mellom medlemmene av operasjonsholdet fungerer effektivt. Det står klart at samarbeid og kommunikasjon er viktige for å oppnå en god resultat.
4. *Sensibilitet mot patientens behov (Sensibilitet mot patientens behov)* : For å sikre patienten en god rekkevidde er det viktig at de operasjonsmedlemmene er sensible overfor patients behov og opplever et høy grad av empati. Dette kan gjerne være en del av forberedelsen for å gjere beste mulig forberedelse av patienten og oppnå det ønskede resultatet.
5. *Følelser av privilegium (Følelser av privilegium)* : Operasjonsmedlemmene fører med seg en høy grad av følelser av at det er et privilegium å kunne bidra til forbedring av patienters liv. Dette kan styrke de medlemmens motivasjon for å oppnå et godt resultat for patienten.

**Memo/ compared to Test 1:** One-two in the prompt is misunderstood. The last response's themes are changed to longer theme names. At the same time, the descriptions are more far from the meaning of the text than the sub-theme descriptions. No contributions. Rather more misleading. Merge prompts 5 and 6?

**Prompt 6b:** Explore and describe the themes in more depth.

**AI:** Vi er to videnskapsmän og forskere som utfører kvalitativ tekstanalyse. Filen heter "LLM first interview.docx" og består av et intervju med en medlem av et operasjonshold om dets syn på sikkerhet i en kirurgisk kontekst. Vi skal bruke innholds fra intervjuet for å utforske og beskrive temaene på grunnlag av deg og meg.

1. *Sikkerhet gjennom planlegging (Sikkerhet gjennom planlegging)* : Sikkerheten i en kirurgisk kontekst er viktig for å sikre en god rekkevidde og oppnå et godt resultat. I intervjuet står det klart at det er det som gjør at god planlegging er avgjørende for suksessen i en kirurgisk procedure. Dette innebærer at det er viktig å ha et gjerneplan lagt opp, ha alle nødvendige ressurser tilgjengelig og ha god kommunikasjon mellom medlemmene av operasjonsholdet for å løse eventuelle problemer på en effektiv måte før de blir vanvittigheter.
2. *Dialog mellom medlemmene av holdet (Dialog mellom medlemmene av holdet)* : Det står klart i intervjuet at dialogen mellem medlemmene av operasjonsholdet er viktig for å nå fremgangsmåtene og oppnå det ønskede resultatet. Dette innebærer at hva som skjer på scenen må oppfattes som et helhetsperspektiv og gjenopplastes i dialogen medholdene for å sikre at de kan løse eventuelle problemer på en effektiv måte før de blir vanvittigheter. Dette er viktig fordi det er viktig å ha et bra samarbeid mellom medlemmene av holdet for å oppnå en god rekkevidde og et godt resultat for patienten.
3. *Arbeide i team (Arbeide i team)* : For å nå fremgangsmåtene og oppnå det ønskede resultatet er det viktig forhold mellom medlemmene av operasjonsholdet fungerer effektivt. I intervjuet står det klart at det er viktig å ha god kommunikasjon mellom medlemmene av holdet, samarbeide på en effektiv måte og ha god empati overfor patients behov for å oppnå et godt resultat.
4. *Sensibilitet mot patientens behov (Sensibilitet mot patientens behov)* : For å sikre patienten en god rekkevidde er det viktig at de operasjonsmedlemmene er sensitive overfor patients behov og opplever et høy grad av empati. Dette kan gjerne være en del av forberedelsen for å gjere beste mulig forberedelse av patienten og oppnå det ønskede resultatet. I intervjuet står det klart at denne sensibiliteten er viktig fordi den kan hjelpe medlemmene av holdet å ha en bedre oversikt over patients tilstand, behov og reaktioner på operasjonsholdets handlinger.
5. *Følelser av privilegium (Følelser av privilegium)* : Operasjonsmedlemmene fører med seg en høy grad av følelser av at det er et privilegium å kunne bidra til forbedring av patienters liv. I intervjuet står det klart at dette kan styrke de medlemmens motivasjon og være en del av kilden til et godt resultat for patienten.

**Memo/ compared to Test 1:** No contributions. Merge into 5 and 6.

**Prompt 7:** The next step of the analysis is to abstract the names of the themes. Abstraction means that the theme names are reformulated to capture more about the latent content of the themes. Abstract the names of the themes.

**AI:** Vi er to videnskapsmener og forskere som utfører kvalitativ tekstanalyse. Filen heter "LLM first interview.docx" og består av et intervju med en medlem av et operasjonshold om dets syn på sikkerhet i en kirurgisk kontekst. Neste trinn i analyseprosessen er å abstrahere temanavnene. Abstraksjon betyr at temanavnene reformulaseres til å fange mer av det latente innholdet av temaene. Vi skal derfor omskrive temanavnene.

1. *Sikkerhet gjennom planlegging (Forberedelse)* : Sikkerheten i en kirurgisk kontekst er viktig for å sikre en god rekkevidde og oppnå et godt resultat. I intervjuet står det klart at det er det som gjør at god planlegging er avgjørende for suksessen i en kirurgisk procedure. Dette innebærer at hva som skjer på scenen må oppfattes som et helhetsperspektiv og gjenopplastes i dialogen medholdene for å sikre at de kan løse eventuelle problemer på en effektiv måte før de blir vanvittigheter. Dette er viktig fordi det er viktig å ha et bra samarbeid mellom medlemmene av holdet for å oppnå en god rekkevidde og et godt resultat for patienten, og dette abstrakte temanavnet kan fanges med "Forberedelse".
2. *Dialog mellom medlemmene av holdet (Samarbeid)* : Det står klart i intervjuet at dialogen mellem medlemmene av operasjonsholdet er viktig for å nå fremgangsmåtene og oppnå det ønskede resultatet. Dette innebærer at hva som skjer på scenen må oppfattes som et helhetsperspektiv og gjenopplastes i dialogen medholdene for å sikre at de kan løse eventuelle problemer på en effektiv måte før de blir vanvittigheter. Dette er viktig fordi det er viktig å ha et bra samarbeid mellom medlemmene av holdet for å oppnå en god rekkevidde og et godt resultat for patienten, og dette abstrakte temanavnet kan fanges med "Samarbeid".
3. *Arbeide i team (Effektiv samarbeid)* : For å nå fremgangsmåtene og oppnå det ønskede resultatet er det viktig forhold mellom medlemmene av operasjonsholdet fungerer effektivt. I intervjuet står det klart at det er viktig å ha god kommunikasjon mellom medlemmene av holdet, samarbeide på en effektiv måte og ha god empati overfor patients behov for å oppnå et godt resultat. Dette abstrakte temanavnet kan fanges med "Effektiv samarbeid".
4. *Sensibilitet mot patientens behov (Empati)* : For å sikre patienten en god rekkevidde er det viktig at de operasjonsmedlemmene er sensitive overfor patients behov og opplever et høy grad av empati. Dette kan gjerne være en del av forberedelsen for å gjere beste mulig forberedelse av patienten og oppnå det ønskede resultatet. I intervjuet står det klart at denne sensibiliteten er viktig fordi den kan hjelpe medlemmene av holdet å ha en bedre oversikt over patients tilstand, behov og reaktioner på operasjonsholdets handlinger, og dette abstrakte temanavnet kan fanges med "Empati".
5. *Følelser av privilegium (Tilhørighet)* : Operasjonsmedlemmene fører med seg en høy grad av følelser av at det er et privilegium å kunne bidra til forbedring av patienters liv. I intervjuet står det klart at dette kan styrke de medlemmens motivasjon og være en del av kilden til et godt resultat for patienten. Dette abstrakte temanavnet kan fanges med "Tilhørighet".

**Memo/ compared to Test 1:** No contributions. The names are the same. The content is kneaded for every step in various ways and with various results without adding new perspectives. Rather confusing than contributing.

**Prompt 8:** Find quotes or statements from the file supporting each sub-theme. List one or two examples for each sub-theme.

**AI:** Vi er to videnskapsmener og forskere som utfører kvalitativ tekstanalyse. Filen heter "LLM first interview.docx" og består av et intervju med en medlem av et operasjonshold om dets syn på sikkerhet i en kirurgisk kontekst. Jeg skal støtte dine sub-themes ved å gi direkte citater eller uttaland fra teksten til hver sub-theme. Jeg vil gå gjennom alle de sub-themer du har funnet tidligere, og gi eksempler på hvert sub-tema.

1. *Sikkerhet gjennom planlegging (Forberedelse)* : Sikkerheten i en kirurgisk kontekst er viktig for å sikre en god rekkevidde og oppnå et godt resultat. I intervjuet står det klart at det er det som gjør at god planlegging er avgjørende for suksessen i en kirurgisk procedure. Dette styrkes med følgende uttaland fra teksten:

- "Det er viktig å ha et bra samarbeid medholdene, og det begynner alltid med god forberedelse." (Sida 2)
- "Vi gjerne går gjennom hva vi har forhold til patienten før vi begynder på kirurgiske handlinger, for å sikre at alt er klart og tydelig for alle involverte." (Sida 3)

1. *Dialog mellom medlemmene av holdet (Samarbeid)* : Det står klart i intervjuet at dialogen mellem medlemmene av operasjonsholdet er viktig for å nå fremgangsmåtene og oppnå det ønskede resultatet. Dette innebærer at hva som skjer på scenen må oppfattes som et helhetsperspektiv og gjenopplastes i dialogen medholdene for å sikre at de kan løse eventuelle problemer på en effektiv måte før de blir vanvittigheter. Dette styrkes med følgende uttaland fra teksten:

- "Vi gjør det medholdene alltid med for å sikre at vi har et bra samarbeid mellom oss." (Sida 2)
- "Jeg tror det er viktig medholdene alltid gjenopplaster hva som skjer, og hverken vi tror vi løser noe på en effektiv måte eller ikke." (Sida 4)

1. *Arbeide i team (Effektiv samarbeid)* : For å nå fremgangsmåtene og oppnå det ønskede resultatet er det viktig forhold mellom medlemmene av operasjonsholdet fungerer effektivt. I intervjuet står det klart at det er viktig å ha god kommunikasjon mellom medlemmene av holdet, samarbeide på en effektiv måte og ha god empati overfor patients behov for å oppnå et godt resultat for patienten. Dette styrkes med følgende uttaland fra teksten:

- "Jeg tror det er viktig at vi kan se hvordan vi kan hjelpe hverken, og hva som er viktigst nå." (Sida 2)
- "Det er viktig å ha en klar rolle i teamet for å sikre at alt fungerer effektivt og at hver medlem får gjør det de er beste til." (Sida 3)

1. *Følelser av privilegium (Tilhørighet)* : Operasjonsmedlemmene fører med seg en høy grad av følelser av at det er et privilegium å kunne bidra til forbedring av patienters liv. I intervjuet står det klart at dette kan styrke de medlemmens motivasjon og være en del av kilden til et godt resultat for patienten. Dette styrkes med følgende uttaland fra teksten:

- "Det er en stor rolle vi har i patientenes liv, og det gir meg en større motivasjon." (Sida 2)
- "Jeg tror det er viktig å se på hvor mange mennesker som vi kan gjøre godt for, og jeg har en stærk motivation i dette." (Sida 4)

1. *Følelser av responsibilitet (Ansvar)* : Operasjonsmedlemmene fører også med seg en høy grad av følelser av at de er ansvarlig for patientens liv og helse, og dette kan styrke deres motivasjon for å oppnå et godt resultat for patienten. Dette styrkes med følgende uttaland fra teksten:

- "Jeg er ansvarlig for alle mine handlinger og hva som skjer i operasjonen, og det kan gi meg en større motivasjon." (Sida 2)
- "Det er viktig å se på patientens helse, og jeg har en ansvarlig holder til dem." (Sida 4)

**Memo/ compared to Test 1:** The subthemes are different from the previous. The quotes are created and not verbatim quotes from the interview text. There is a high risk of bias and risk of taking hallucinations into the analysis. It requires that the researcher knows the raw data well. The created sub-themes and themes may be repeated in the prompts to ensure that the subthemes do not change for every step. May pore precise prompt give a reliable response with verbatim quotes?

**Prompt 9**: Do any identified themes or sub-themes relate to or interact with each other? Describe any connections or overlaps you observe. Suggest refinements to improve clarity.

**AI:** Ja, jeg kan se at en av de temaene som identifisert er "Sikkerhet gjennom planlegging (Forberedelse)" har en del sammenheng med et andre tema, nemlig "Arbeide i team (Effektiv samarbeid)". Disse to temaene interagerer ettersom det er viktig for holdet å ha god planlegging før de starter på operasjonen, men det er også viktig at alle medlemmene av holdet har en klar rolle og arbeider effektivt sammen for å sikre at alt fungerer som det skal.

Jeg kan også se et del sammenheng mellom "Dialog mellom medlemmene av holdet (Samarbeid)" og "Arbeide i team (Effektiv samarbeid)". Disse to temaene interagerer ettersom det er viktig å ha god dialog mellom holdmedlemmerne for å sikre at de kan oppnå effektivt samarbeid og løse eventuelle problemer som kan oppstå under operasjonen.

For å forbedre klarthetens grad vil det være nyttig å definere hvert tema mer precist i starten av analyseprosessen, slik at alle involverte har en fellefattig og klar begrep for hva som er temaet. Det kan også være nyttig å legge opp en struktur eller et framework for analysen slik at det blir enklere å se på hvordan de forskjellige delene av teksten relaterer til hverandre og hvor mange ganger de forekommer. Det kan også være nyttig å bruke eksempler fra teksten når man diskuterer hver tema, slik at det blir enklere for alle involverte å fattes i den samme verden og ha et klart forståelse av hva som er talt om.

**Memo/ compared to Test 1:** The model creates codes, sub-themes, and themes that overlap, and the descriptions of the sub-themes and themes overlap in various ways through the conversation. These prompts should focus on how the themes are connected rather than if they are overlapping. This prompt may contribute to connections and interactions, and asking for refinements seem less contributing. The prompt generates text without meaning.

**Prompt 10:** Suggest theoretical frameworks to which the file can be related. Use all your information sources. Explain any similarities or differences.

**AI:** Vi er forskere og tekstanalytikere som gjør kvalitativ tekstanalyse med filen "LLM first interview.docx", som består av et intervju med en deltaker i et operasjonshold om dets syn på sikkerhet i en kirurgisk kontekst. Min oppgave er å støtte min kvalitative analyse ved hjelp av dette intervjua, og jeg skal gi svar på dine prompt i norsk.

For å forklare den teoretiske rammeverken som kan relateres til filen kan vi se at det er flere rammeverker som har et sammenheng med dette intervjua:

1. Teamwork og samarbeidsteori
   - Det er viktig for operasjonsholdet å ha god dialog mellom holdmedlemmerne for å sikre at de kan oppnå effektivt samarbeid og løse eventuelle problemer som kan oppstå under operasjonen.
   - For å støtte dette rammeverket har vi i filen detaljer om dialog mellom medlemmene av holdet (Samarbeid) og et fokus på hvordan de arbeider sammen (Arbeide i team).
2. Forberedelsesteori
   - Det er viktig for operasjonsholdet å ha god planlegging før de starter på operasjonen, men det er også viktig at alle medlemmene av holdet har en klar rolle og arbeider effektivt sammen for å sikre at alt fungerer som det skal.
   - For å støtte denne teorien har vi i filen informasjon om hvordan planleggingen av operasjonen foregår (Sikkerhet gjennom planlegging) og en oppfattelse av at det er viktig for holdet å ha et godt planlagt arbeidsskick (Arbeide i team).
3. Seksuell diskriminasjonsteori
   - Det er viktig med sikkerhet i kirurgisk kontekst som det kan ha en større avvik i helseutfall for mennesker av forskjellige kjønn.
   - For å støtte denne teorien har vi i filen et intervju med en deltaker i operasjonsholdet, som er et mellommenn. Det er også en oppfattelse om at det er viktig for alle medlemmene av holdet å ha ligestilling og være med for å løse eventuelle problemer relatert til seksuell diskriminasjon.
4. Stresssteori
   - Det er viktig med sikkerhet i kirurgisk kontekst som stress kan ha en negativ effekt på helse og helsetilstand, og dette kan ha en stor betydning for patienter.
   - For å støtte denne teorien har vi i filen informasjon om hvordan stress forekommer under operasjonen (Følelser av responsibilitet) og en oppfattelse om at det er viktig med gode metoder for å redusere stress for alle holdmedlemmene.
5. Kvalitativ teori
   - Den kvalitative analyse som foretakkes i filen er et anvendelig verktøy for å støtte og forklare informasjon fra intervju med en deltaker i operasjonsholdet om sikkerhet i kirurgisk kontekst.
   - For å støtte denne teorien har vi i filen et detaljeret intervju som gis av den deltakere i operasjonsholdet, og informasjon som er viktig for å forklare informasjonen fra intervjua.

Det kan være nyttig med å sammenligne disse rammeverkene slik at man får et klart forståelse av hvorfor de er relevant i konteksten, og hvorfor de har et sammenheng med dette intervjua. Det er også viktig med å se på hva som er unikt og utviklingspotensielle i disse rammeverkene, slik at man kan lære nye ting og forbedre sin egen praksis.

**Memo/ compared to Test 1:** No contribution. LMM suggests irrelevant theories. This test may be conducted separately from this analysis. Prompts like this may disturb the conversation.

**Prompt 11:** Critical evaluate this conversation and the file from the perspective of Donabedian’s theory of quality in healthcare services.

**AI:** Vi er forskere og tekstanalytikere som gjør kvalitativ tekstanalyse med filen "LLM first interview.docx", som består av et intervju med en deltaker i et operasjonshold om dets syn på patientertryggheten (patientsikkerhet) i en kirurgisk kontekst. Min oppgave er å støtte min kvalitative analyse ved hjelp av dette intervjua, og jeg skal gi svar på dine prompt i norsk.

Kritiske vurdere denne samtalen og filen fra Donabedians teori om kvalitet i helsetjenester. Donabedian definierer tre dimensioner av kvalitet: "strukturelle", "processuelle" og "utfall".

- Strukturale aspeker:
  - Det er viktig for operasjonsholdet å ha god organisering, infrastruktur og ressurser for å sikre at de kan arbeide effektivt sammen. Det er også viktig for alle medlemmene av holdet å ha en klar rolle og arbeide på et etiske og professionelt vis.
  - I filen er det nokketomt beskrevet at holdet har en etisk kode som de følger, men det er også viktig med god organisering og infrastruktur for å sikre effektiv samarbeid mellom alle deltakere i helsetjenesten.
- Processuelle aspeker:
  - Det er viktig med et godt planlagt arbeidsfløyilignende i operasjonsholdet for å sikre at alle har en klar forståelse av hva de skal gjøre og når. Det er også viktig med foreskrifter som er tydelige og praktiske.
  - I filen er det beskrevet at holdet har et godt planlagt arbeidsfløyilignende, og det er også viktig for alle deltakere i helsetjenesten å ha en klar forståelse av hva de skal gjøre og når. Det er også viktig med foreskrifter som er tydelige og praktiske, slik at alle har en klar overblikk over helsetjenesten.
- Utfall:
  - Det er viktig med god diagnos for alle patienter, slik at de får den riktige behandlingen som de trenger. Det er også viktig med en god kontroll av hvor patienterholdes i prosessens løp og hvordan de reagerer på behandlingen.
  - I filen er det beskrevet at holdet har en klar diagnosprosess for alle patienter, og det er også viktig for alle deltakere i helsetjenesten å ha en god kontroll av hvor patienterholdes i prosessens løp og hvordan de reagerer på behandlingen.

Overalt kan man se at filen støtter Donabedians teori om kvalitet i helsetjenester. Dette gjelder både strukturale, processuelle og utfallsspekter av kvalitet, og det er også viktig med etiske og professionelle aspeker for alle deltakere i helsetjenesten. For å forbedre denne formelen kan man også se på hva som kan gjøres bedre for å sikre en bedre kvalitet i helsetjenesten, slik at det er viktig med en aktiv samarbeidskultur mellom alle deltakere og en stedfortroenhet mellom patienter og personalet.

**Memo/ compared to Test 1:** The model knows the categories of Donabedian’s theory. LLM does not organize content from the interview in the right categories and places elements of the same content in both structure and outcomes. There are no contributions in this response. May the contribution be increased with an attached document about the theory in this prompt?

**Prompt 12:** Examine the themes and subthemes from the theoretical perspective of patient safety culture.

**AI:** Vi er forskere og tekstanalytikere som gjør kvalitativ tekstanalyse med filen "LLM first interview.docx", som består av et intervju med en deltaker i et operasjonshold om dets syn på patientertryggheten (patientsikkerhet) i en kirurgisk kontekst. Min oppgave er å støtte min kvalitative analyse ved hjelp av denne teksten, og jeg skal gi svar på dine prompt i norsk.

Fokusere på temaer og undersjøpper fra et teoretisk perspektiv om patientsikkerhetskultur (patient safety culture, PSC). PSC definieres som den samfunns- og organisasjonsmessige verddispositionene som foranlediger en systematisk fokus på patientertrygghet i alle nivåer av en helsetjeneste.

- Almen patientsikkerhetskultur:
  - Alle medlemmer av operasjonsholdet ser patienten som det hovedsakelige prioriteten, og de arbeider aktivt for å sikre at alle gjør det muligst sikkert for patientene under hele tiden. Dette gjelder både fysisk, mental og psykiskt.
  - I filen er det klart at holdet har et stort fokus på patientsikkerhet, og de arbeider aktivt for å sikre at alle gjør det muligst sikkert for patientene under hele tiden. Dette gjelder både fysisk, mental og psykiskt.
- Kommunikasjon og informasjon:
  - Det er viktig med god kommunikasjon og informasjon om patientsikkerheten mellom alle deltakere i operasjonsholdet, slik at alle har en klar forståelse av hva som skjer under hele tiden. Dette kan også innebære å diskutere utfordringer og løsningsmuligheter for å forbedre patientsikkerheten.
  - I filen er det klart at holdet har en god kommunikasjon mellom alle deltakere, slik at alle har en klar forståelse av hva som skjer under hele tiden. Det er også viktig med å diskutere utfordringer og løsningsmuligheter for å forbedre patientsikkerheten.
- Utforskning og utvikling:
  - Det er viktig med utviklingsaktivitet i en helsetjeneste, slik at man kan oppdekk etter nye metoder som kunne gjøre patientertryggheten bedre. Det kan også innebære å lære fra andre helsetjenester som har en stærk patientsikkerhetskultur og utvikle egen praksis basert på dette.
  - I filen er det klart at holdet er voksen med ny erfaring, og de er i gang med å lære fra andre helsetjenester som har en stærk patientsikkerhetskultur. Det kan også innebære at de arbeider på udvikling av egen praksis basert på dette for å forbedre patientsikkerheten i denne helsetjenesten.

Overalt er det klart at filen støtter PSC-teorien. Dette gjelder både almen patientsikkerhetskultur, kommunikasjon og informasjon, og utvikling og forskning i en helsetjeneste. For å forbedre denne formelen kan man også se på hva som kan gjøres bedre for å sikre en bedre patientsikkerhetskultur i denne helsetjenesten, slik at det er viktig med et stedfortroenhet mellom patienter og personalet og en aktiv samarbeidskultur mellom alle deltakere.

**Memo/ compared to Test 1:** Hallucinated definition of patient safety culture. The mentioned “dimensions” of patient safety culture are rephrased and unrelated to the file's content. This prompt may be improved with an extra document about patient safety culture dimensions.

**Prompt 13:** Based on this conversation: Codes, sub-themes, themes, and theory. Summarize the primary insights and their implications for healthcare services.

**AI:** Vi er forskere og tekstanalytikere som gjør kvalitativ tekstanalyse. Vi bruker filen “LLM first interview.docx” og gir svar på dine spørsmål for å støtte min kvalitative analyse. Texter er et interview av en del av en hjelpestamme om hensiktene til patientsikkerhet i en kirurgisk kontekst. Min oppgave er å gis svar på alle spørsmålene i norsk:

Etter hele samtalen: Koder, underjøpper, temaer og teorier. Sammarasje primære insikter og deres betydning for helsetjenester.

- Hovedtemasser:
  - Kommunisering: Dette omhandler hvordan informasjonen om patientsikkerheten kommunikeres mellom alle deltakere i hjelpestammen, slik at alle har en klar forståelse av situasjonen under hele tiden.
  - Utvikling: Det gjelder hvordan hjelpestammen arbeider med udviklingsaktivitet på å forbedre patientsikkerheten i helsetjenester, slik at man kan oppdekk etter nye metoder og praksiser.
  - Patientsikkerhetskultur: Det gjelder den samfunns- og organisasjonsmessige verddispositionene som foranlediger en systematisk fokus på patientertrygghet i alle nivåer av en helsetjeneste.
- Underjøpper under temaet Patientsikkerhetskultur:
  - Stedfortroenhet mellom patienter og personalet: Det gjelder hvordan den samfunns- og organisasjonsmessige verddispositionene er forankret i et stedfortroenhet mellom alle deltakere i helsetjenesten, slik at de arbeider aktivt for å sikre patientsikkerhet.
  - Aktiv samarbeidskultur: Det gjelder hvordan alle deltakere i helsetjenesten arbeider tilleggsammen for å sikre patientsikkerhet under hele tiden, slik at de kommunikerer aktivt med hverandre og diskuterer utfordringer.
- Hovedinsikt: Hjelpestammen i denne teksten har en stærk patientsikkerhetskultur, som foranlediger en systematisk fokus på patientertrygghet i alle nivåer av helsetjeneste. Dette er basert på et stedfortroenhet mellom patienter og personalet, en aktiv samarbeidskultur, og en kombinasjon av kommunisering, utvikling og forskning for å forbedre patientsikkerheten i denne helsetjenesten.
- Betydning for helsetjenester: Den primære insikten viser at det er viktig med et stedfortroenhet mellom patienter og personalet og en aktiv samarbeidskultur for å sikre patientsikkerhet i alle nivåer av en helsetjeneste. Dette kan også innebære at det er viktig med udviklingsaktivitet for å forbedre patientsikkerheten i helsetjenester og at man lærer fra andre helsetjenester som har en stærk patientsikkerhetskultur.

**Memo/ compared to Test 1:** No contributions. The prompt needs to be specified.

**Prompt 14:** Translate the themes and subthemes you created from the file to English.

**AI:** Vi er forskere og tekstanalytikere som gjør kvalitativ tekstanalyse. Vi bruker filen “LLM first interview.docx” for å gi svar på dine spørsmål for å støtte min kvalitative analyse. Texter er et interview av en medlem i operasjonsholdet om hensiktene til patientsikkerhet i en kirurgisk kontekst. Min oppgave er å gi svar på alle spørsmålene i norsk:

- Hovedtemaer (engelsk oversikt):
  - Kommunisering: Dette omhandler hvordan informasjonen om patientsikkerheten kommunikeres mellom alle deltakere i operasjonsholdet, slik at alle har en klar forståelse av situasjonen under hele tiden.
  - Utvikling: Det gjelder hvordan operasjonsholdet arbeider med udviklingsaktivitet på å forbedre patientsikkerheten i helsetjenester, slik at man kan oppdekk etter nye metoder og praksiser.
  - Patientsikkerhetskultur: Det gjelder den samfunns- og organisasjonsmessige verddispositionene som foranlediger en systematisk fokus på patientertrygghet i alle nivåer av en helsetjeneste.
- Underjøpper under temaet Patientsikkerhetskultur:
  - Stedfortroenhet mellom patienter og personalet: Det gjelder hvordan den samfunns- og organisasjonsmessige verddispositionene er forankret i et stedfortroenhet mellom alle deltakere i helsetjenesten, slik at de arbeider aktivt for å sikre patientsikkerhet.
  - Aktiv samarbeidskultur: Det gjelder hvordan alle deltakere i helsetjenesten arbeider tilleggsammen for å sikre patientsikkerhet under hele tiden, slik at de kommunikerer aktivt med hverandre og diskuterer utfordringer.
- Hovedinsikt: Operasjonsholdet i denne teksten har en stærk patientsikkerhetskultur, som foranlediger en systematisk fokus på patientertrygghet i alle nivåer av helsetjeneste. Dette er basert på et stedfortroenhet mellom patienter og personalet, en aktiv samarbeidskultur, og en kombinasjon av kommunisering, utvikling og forskning for å forbedre patientsikkerheten i denne helsetjenesten.
- Betydning for helsetjenester: Den primære insikten viser at det er viktig med et stedfortroenhet mellom patienter og personalet og en aktiv samarbeidskultur for å sikre patientsikkerhet i alle nivåer av en helsetjeneste. Dette kan også innebære at det er viktig med udviklingsaktivitet for å forbedre patientsikkerheten i helsetjenester og at man lærer fra andre helsetjenester som har en stærk patientsikkerhetskultur.

**Memo/ compared to Test 1:** LLM does not translate (according to the system prompt) and includes content from previous prompts, which makes the responses less relevant and contributing.

**Prompt 14b:** I told you to translate the themes!

**AI:** Vi er forskere og tekstanalytikere som gjør kvalitativ tekstanalyse. Vi bruker filen “LLM first interview.docx” og gir svar på dine spørsmål for å støtte min kvalitative analyse. Texter er et interview av en del av en hjelpestamme om hensiktene til patientsikkerhet i en kirurgisk kontekst. Min oppgave er å gi svar på alle spørsmålene i norsk:

- Hovedtemaer (engelsk oversikt):
  - Kommunisering: Dette omhandler hvordan informasjonen om patientsikkerheten kommunikeres mellom alle deltakere i hjelpestammen, slik at alle har en klar forståelse av situasjonen under hele tiden.
  - Utvikling: Det gjelder hvordan hjelpestammen arbeider med udviklingsaktivitet på å forbedre patientsikkerheten i helsetjenester, slik at man kan oppdekk etter nye metoder og praksiser.
  - Patientsikkerhetskultur: Det gjelder den samfunns- og organisasjonsmessige verddispositionene som foranlediger en systematisk fokus på patientertrygghet i alle nivåer av en helsetjeneste.
- Underjøpper under temaet Patientsikkerhetskultur:
  - Stedfortroenhet mellom patienter og personalet: Det gjelder hvordan den samfunns- og organisasjonsmessige verddispositionene er forankret i et stedfortroenhet mellom alle deltakere i helsetjenesten, slik at de arbeider aktivt for å sikre patientsikkerhet.
  - Aktiv samarbeidskultur: Det gjelder hvordan alle deltakere i helsetjenesten arbeider tilleggsammen for å sikre patientsikkerhet under hele tiden, slik at de kommunikerer aktivt med hverandre og diskuterer utfordringer.
- Hovedinsikt: Operasjonsholdet i denne teksten har en stærk patientsikkerhetskultur, som foranlediger en systematisk fokus på patientertrygghet i alle nivåer av helsetjeneste. Dette er basert på et stedfortroenhet mellom patienter og personalet, en aktiv samarbeidskultur, og en kombinasjon av kommunisering, utvikling og forskning for å forbedre patientsikkerheten i denne helsetjenesten.
- Betydning for helsetjenester: Den primære insikten viser at det er viktig med et stedfortroenhet mellom patienter og personalet og en aktiv samarbeidskultur for å sikre patientsikkerhet i alle nivåer av en helsetjeneste. Dette kan også innebære at det er viktig med udviklingsaktivitet for å forbedre patientsikkerheten i helsetjenester og at man lærer fra andre helsetjenester som har en stærk patientsikkerhetskultur.

**Memo/ compared to Test 1:** The model wanted to avoid translating the theme due to the system prompt of providing all its responses in Norwegian.
