# Supplementary material for: Reflecting on LLM Support in Reflexive Thematic Analysis: An Exploratory Study
Source: Qual Health Res. 2025 Sep 8;36(2-3):191–205. doi: 10.1177/10497323251365211 (PMC12949038; doi:10.1177/10497323251365211)
Supplement: Supplemental Material - Reflecting on LLM Support in Reflexive Thematic Analysis: An Explorative Study [file sj-zip-1-qhr-10.1177_10497323251365211.zip › Supplemental_File_8_Test_5b.docx]

Ollama Mistral Test 5b- translated text document and responses in English (language settings: English-GB). Added an extra document on Donabedian`s theory.

System prompt: You and I are scientists and researchers doing qualitative text analysis. The text is an interview of a surgical team member's perspectives on patient safety in a surgical context.

Prompt 1: Use the file on Donabedian’s theory of quality in healthcare services and examine the file “LLM first interview translated for analysis“ in Donabedian`s framework.

**AI:**

According to Donabedian's 1966 paper Donabedian 1966.pdf, his theory of quality in healthcare services focuses on three dimensions: structure, process, and outcome.

In terms of the "LLM first interview translated for analysis" LLM first interview translated for analysis.docx, we can analyze it using Donabedian's framework as follows:

1. Structure: The interviewee talks about the working conditions, including wages, work environment, and management. They also mention the need for experienced staff for continuity and safety in patient care, which highlights the importance of having a well-organized structure to provide quality healthcare services.
2. Process: The interviewee discusses the process of treating patients, such as the time spent on each patient, ensuring patient safety, and balancing efficiency with patient care. They also mention the dialogue between professionals and management about various issues affecting their work environment at the hospital.
3. Outcome: Although not explicitly mentioned in the interview, the outcome could be inferred from the interviewee's concerns for patient safety and well-being. The interviewee highlights that efficient treatment does not always mean increased output or income; instead, it emphasizes ensuring patient safety and quality of care.

Overall, the "LLM first interview translated for analysis" can be analyzed using Donabedian's framework by considering its structure (hospital working conditions), process (treatment approach and dialogue between professionals and management), and outcome (patient safety and well-being). The interviewee emphasizes the importance of balancing efficiency with patient care, which is in line with Donabedian's theory of quality in healthcare services.

**Memo/ compared to Test 5a:**

This test and prompt provide a response that supports the analysis better than including this in the analysis process. Structural and processual factors display content from the interview and add perspectives not mentioned in test 5a.

Prompt 1: Could you explore the interview text more in details?

AI:

To provide a more detailed exploration of the interview text, I will first highlight some key points and then discuss them further in terms of their relevance to patient safety in a surgical context using the Donabedian's framework. Here is the excerpt from the interview:

Interviewer: What are your thoughts on patient safety during surgeries?

Respondent: First, it's essential that the team communicates effectively and maintains open dialogue to avoid misunderstandings or errors. Second, proper equipment and tools must be available and in good working order. Third, all team members should have a clear understanding of their roles and responsibilities, as well as the overall plan for the surgery. Fourth, we should always strive to minimize invasiveness while achieving the desired outcome. Lastly, we need to remember that patient safety is not only about technical skills but also about empathy, respect, and compassion towards the patient.

Now let's analyze this excerpt using Donabedian's framework:

1. Structure: The respondent mentions the importance of proper equipment and tools (structure). A well-maintained infrastructure supports safe surgical practices. Additionally, having a clear understanding of roles and responsibilities contributes to an effective organizational structure.
2. Process: The emphasis on open communication, maintaining dialogue, and minimizing invasiveness demonstrates the importance of good processes during surgery. Effective communication reduces misunderstandings and errors that could compromise patient safety. Minimizing invasiveness while achieving desired outcomes balances efficiency with quality care.
3. Outcome: The respondent acknowledges that patient safety is not only about technical skills but also empathy, respect, and compassion towards the patient (outcome). This shows an understanding of the emotional and psychological aspects of patient care, which contribute to a positive outcome for the patient.

By using Donabedian's framework, we can see how the interview text highlights the importance of structure, process, and outcome in ensuring patient safety during surgeries. The respondent emphasizes the role of effective communication, appropriate equipment, clear roles and responsibilities, minimizing invasiveness, and empathy, respect, and compassion towards patients as crucial factors for enhancing patient safety in a surgical context.

**Memo/ compared to Test 5a:**

LLM creates an interview text/ quotation. This response is mainly based on sources other than the interview file.
