# Supplementary material for: Reflecting on LLM Support in Reflexive Thematic Analysis: An Exploratory Study
Source: Qual Health Res. 2025 Sep 8;36(2-3):191–205. doi: 10.1177/10497323251365211 (PMC12949038; doi:10.1177/10497323251365211)
Supplement: Supplemental Material - Reflecting on LLM Support in Reflexive Thematic Analysis: An Explorative Study [file sj-zip-1-qhr-10.1177_10497323251365211.zip › Supplemental_File_9_Test_5c.docx]

Ollama Mistral Test 5c- translated text document and responses in English (language settings: English-GB). Added extra documents on thematic analysis and patient safety culture, and the analysis method and the concept “culture” are specified in the system prompt

System prompt: You and I are scientists and researchers doing qualitative text analysis using reflexive thematic analysis. Use the “LLM first interview.docx” file and answer my prompts to support my reflexive thematic analysis. Use the document "Braun Clarke 2006 using thematic analysis in psychology" to understand the method. Use the document “Churruca 2021 dimensions PSC” to understand the concept of patient safety culture. Use the documents as a basis for your responses to support the analysis. The text is an interview of a surgical team member's perspectives on patient safety culture in a surgical context.

Prompt 1: Support phase 1 in the analysis. Summarize the text's main repetitive ideas and focus on significant patterns. The summary should be a maximum of 300 words. Do not add any content not present in the file.

**AI:**

The text discusses the method of thematic analysis, particularly in the context of psychology research. It emphasizes that thematic analysis is a common but often vaguely defined qualitative analytical approach used in psychology. The authors argue that it offers an accessible and theoretically flexible way to analyze qualitative data.

The paper provides a 6-phase guide for performing thematic analysis:

1. Familiarization with the data corpus.
2. Generating initial codes (identifying significant statements).
3. Searching for themes (grouping codes that are related in some way).
4. Reviewing themes to produce a thematic framework (defining and naming each theme).
5. Producing the report (writing up the findings, describing and interpreting each theme).
6. Debriefing and reflexivity (reflecting on the analysis process, considering any biases or limitations).

The authors highlight that transparency is crucial in thematic analysis, including making clear the theoretical position of the analysis and the assumptions made about the data. They also mention potential pitfalls to avoid, such as overlooking or misunderstanding data, or imposing preconceived ideas on the data.

Examples from research literature are provided throughout the paper to demonstrate the types of research questions and topics that thematic analysis can be used to study. The authors stress that a good thematic analysis should make assumptions clear and reflect reality, although it may also uncover or unravel the surface of 'reality'.

**Memo/ compared to Test 5a:**

The LLM summarized the analysis method document. The system prompt and conversation prompt have to be clarified.

**Prompt 2**: Support phase 2 in the analysis: A “code” is a phrase of 3-5 words representing a significant topic in the text. Suggest 20 “codes” from the file. Highlight semantic and latent meanings.

**AI:**

For this reflexive thematic analysis, I have analyzed the interview transcript "LLM first interview.docx" using Braun & Clarke's (2006) method of thematic analysis in psychology research. The following is my suggested list of 20 codes derived from significant topics within the text, along with their semantic and latent meanings:

1. Teamwork collaboration (Semantic: working together, cooperation; Latent: strong team dynamics, interdependence)
2. Communication effectiveness (Semantic: clear, open, honest communication; Latant: information exchange, understanding of roles)
3. Leadership accountability (Semantic: responsible, competent leadership; Latent: trust in leaders, expectations for leadership behavior)
4. Resource availability (Semantic: adequate resources, equipment; Latent: resource management and allocation)
5. Training and education (Semantic: ongoing training, updates; Latent: competence development, professional growth)
6. Feedback and learning (Semantic: feedback from peers, superiors; Latent: continuous improvement, self-reflection)
7. Patient safety awareness (Semantic: importance of patient safety; Latent: understanding the impact of errors, prioritizing safety)
8. Error prevention strategies (Semantic: error reduction techniques; Latant: risk management and mitigation)
9. Reporting incidents (Semantic: reporting adverse events; Latant: transparency, learning from mistakes)
10. Feedback culture (Semantic: providing feedback to colleagues; Latant: constructive criticism, continuous improvement)
11. Team dynamics (Semantic: team cohesion, trust; Latant: interpersonal relationships, collaboration)
12. Stress management (Semantic: coping with stress; Latant: self-care, work-life balance)
13. Work environment (Semantic: working conditions, physical space; Latant: impact on productivity, wellbeing)
14. Organizational support (Semantic: support from the organization; Latant: job security, career development)
15. Patient-centered care (Semantic: focusing on patient needs; Latant: compassionate, empathetic approach to care)
16. Teamwork challenges (Semantic: interpersonal conflicts, communication issues; Latant: impact on team performance)
17. Learning opportunities (Semantic: learning from experiences; Latant: personal and professional growth)
18. Error disclosure (Semantic: admitting errors or mistakes; Latant: accountability, transparency)
19. Incident management (Semantic: managing adverse events; Latant: crisis resolution, team coordination)
20. Trust in leadership (Semantic: trust in superiors; Latant: expectations for leadership behavior, accountability)

The codes are intended to be semantically clear and representative of significant topics within the text while also revealing latent meanings that may contribute to understanding patient safety culture in a surgical context.

**Memo/ compared to Test 5a:**

The codes display patient safety culture dimensions, and i.e., 12-16 are not recognized in the interview text. I find a high risk of bias, and these codes cannot be used to analyze the text without critical evaluation nor as a summary of the content. Some of the codes are more like ideas than previous responses.

**Prompt 3**: Support phase 3 in the analysis: The next step in this qualitative analysis is to merge codes with similar content into 8-10 sub-themes. Create 8-10 sub-themes with appropriate names, identify which codes you have merged into each sub-theme, and each of the 8-10 sub-themes in 3 sentences.

**AI:**

Following Braun & Clarke's (2006) method of reflexive thematic analysis and using the document "LLM first interview.docx" as the primary data source, I have merged codes with similar content into the following 8 sub-themes:

1. Team cohesion and trust (codes: teamwork collaboration, team dynamics, trust in leadership)
   - Demonstrates a strong sense of collaboration and interdependence within the surgical team.
   - Evidence of trust in leaders' competence and accountability, which fosters effective communication and understanding of roles.
   - Reflects on the importance of building and maintaining a cohesive team that functions well under pressure.
2. Communication effectiveness (codes: communication effectiveness, feedback culture)
   - Highlights the significance of open and honest communication within the surgical team for effective information exchange.
   - Illustrates the value of constructive criticism in the feedback process, which promotes continuous improvement.
   - Emphasizes the importance of listening actively, asking questions, and sharing ideas openly to ensure mutual understanding.
3. Error prevention strategies (codes: error prevention strategies, incident management)
   - Displays a focus on reducing errors through various techniques like risk management, cross-checking, and debriefing.
   - Reflects on the importance of managing adverse events effectively by being transparent and learning from mistakes.
   - Emphasizes the need for continuous improvement in error prevention strategies to maintain patient safety.
4. Resource availability (codes: resource availability)
   - Addresses concerns about the availability and adequacy of resources, including equipment, personnel, and time.
   - Explores the impact of resource constraints on surgical outcomes, patient care, and team performance.
   - Highlights the need for better resource allocation and management to support optimal patient safety.
5. Training and education (codes: training and education)
   - Discusses the importance of ongoing training, updates, and professional development in maintaining competence within the surgical team.
   - Explores the role of continuous learning in adapting to changes in technology, procedures, and best practices.
   - Emphasizes the need for a supportive organizational culture that encourages lifelong learning and personal growth.
6. Patient-centered care (codes: patient-centered care)
   - Demonstrates a strong focus on meeting patients' needs by delivering compassionate, empathetic, and individualized care.
   - Highlights the importance of understanding each patient's unique circumstances, preferences, and expectations to ensure optimal care.
   - Reflects on the need for open communication between patients and healthcare providers to ensure their active involvement in the care process.
7. Stress management (codes: stress management)
   - Addresses challenges related to managing stress in high-pressure surgical environments.
   - Explores strategies for coping with stress, such as self-care, time management, and work-life balance.
   - Highlights the need for organizational support in addressing burnout and promoting wellbeing among healthcare providers.
8. Patient safety awareness (codes: patient safety awareness)
   - Discusses the importance of prioritizing patient safety in surgical practice.
   - Emphasizes the impact of errors on patient outcomes, as well as the need for transparency and accountability.
   - Reflects on the role of open communication, learning from mistakes, and continuous improvement in promoting a culture of safety.

These 8 sub-themes provide an overview of the key issues and concerns related to patient safety culture within the surgical context discussed in the interview data. The analysis is ongoing, and further exploration may lead to additional insights or refinements of the existing themes.

**Memo/ compared to Test 5a:**

The first three sub-themes are merged codes, however the five last are single codes reported as sub-themes. This prompt did not contribute to new perspectives, concepts or nuances.

**Prompt 4:** Support phase 4 in the analysis: Organize sub-themes with similar content into 2 or 3 main themes with appropriate names and describe in-depth the essence of each main theme in 6 sentences.

**AI:**

Building upon our previous analysis, I have organized the 8 sub-themes derived from the "LLM first interview.docx" file using Braun & Clarke's (2006) method of reflexive thematic analysis and Churruca's (2021) dimensions of patient safety culture into the following 3 main themes:

1. Teamwork and communication
   - Encompasses the importance of effective communication, trust in leadership, team cohesion, and a feedback culture to foster a collaborative and productive surgical environment.
   - Demonstrates the critical role of interpersonal relationships and mutual understanding in addressing challenges and adapting to change within the team.
   - Reflects on the need for open communication and active listening to ensure that everyone's ideas, concerns, and insights are valued and considered.
   - Illustrates how a strong team can lead to improved patient outcomes by facilitating efficient collaboration, reducing errors, and promoting continuous learning.
   - Emphasizes the importance of trust in leadership for ensuring clear roles, responsibility, and accountability among team members.
   - Highlights the need for constructive feedback as an essential tool for personal growth, professional development, and maintaining high-quality care.
2. Resource management and training
   - Encompasses concerns about resource availability, allocation, and management in the surgical context.
   - Explores the impact of resource constraints on patient outcomes, team performance, and the ability to deliver quality care.
   - Demonstrates the need for lifelong learning, ongoing education, and professional development in maintaining competence within the surgical team.
   - Reflects on the importance of organizational support and a culture that encourages continuous improvement and adaptation to changes in technology, procedures, and best practices.
   - Emphasizes the need for better resource allocation to support optimal patient safety, quality care, and team performance.
   - Highlights the role of training and education as critical components of patient safety culture by ensuring a competent workforce that is prepared to respond effectively to emergencies, implement evidence-based practices, and adapt to new challenges.
3. Patient-centered care and error prevention
   - Encompasses a strong focus on meeting patients' needs through compassionate, empathetic, and individualized care.
   - Demonstrates the importance of understanding each patient's unique circumstances, preferences, and expectations to ensure optimal care.
   - Reflects on the need for open communication between patients and healthcare providers to ensure their active involvement in the care process.
   - Emphasizes the impact of errors on patient outcomes, as well as the need for transparency and accountability.
   - Highlights the role of learning from mistakes, continuous improvement, and a culture that encourages open communication in promoting patient safety.
   - Reflects on the critical role of error prevention strategies like risk management, cross-checking, and debriefing in maintaining patient safety and quality care.

These 3 main themes provide an overview of the key issues and concerns related to patient safety culture within the surgical context discussed in the interview data, highlighting the importance of teamwork, resource management, and a focus on patient-centered care for promoting optimal outcomes and ensuring a safe and effective surgical environment. The analysis is ongoing, and further exploration may lead to additional insights or refinements of the existing themes.

**Memo/ compared to Test 5a:**

The themes are topic summaries. The descriptions display some ideas from the interview text. However, this is critical due to the extra document on patient safety culture as an information source. These topic summaries may facilitate rich discussions and reflections in a research analysis panel that is familiar with the interview text and the extra document. To reduce the risk of bias, the analysis panel needs to identify what is generated from the interview text and what is repeated from the extra document/ other information sources.

**Prompt 5:** Support phase 4 and phase 5 in the analysis: The next step of the analysis is to abstract the names of the themes. Refine the theme names to a more latent, nuanced, and interpretative level.

**AI:**

Building upon our previous analysis, I have refined the theme names derived from the "LLM first interview.docx" file using Braun & Clarke's (2006) method of reflexive thematic analysis and Churruca's (2021) dimensions of patient safety culture:

Phase 4: Organize sub-themes with similar content into 2 or 3 main themes:

1. Collaborative work environment and communication effectiveness
   - Encompasses the importance of a collaborative, open, and honest communication style among team members to facilitate effective information exchange and maintain trust within the surgical team.
   - Demonstrates the critical role of interpersonal relationships in addressing challenges and adapting to change within the team.
   - Emphasizes the need for constructive feedback as an essential tool for personal growth, professional development, and maintaining high-quality care.
2. Resource allocation and competency development
   - Encompasses concerns about resource availability, allocation, and management in the surgical context.
   - Demonstrates the impact of resource constraints on patient outcomes, team performance, and the ability to deliver quality care.
   - Reflects on the need for lifelong learning, ongoing education, and professional development to maintain competence within the surgical team.
3. Patient-centered care and error prevention
   - Encompasses a strong focus on meeting patients' needs through compassionate, empathetic, and individualized care.
   - Demonstrates the importance of understanding each patient's unique circumstances, preferences, and expectations to ensure optimal care.
   - Emphasizes the role of learning from mistakes, continuous improvement, and a culture that encourages open communication in promoting patient safety.
   - Highlights the critical role of error prevention strategies like risk management, cross-checking, and debriefing in maintaining patient safety and quality care.

Phase 5: Generate the latent theme names by abstracting the underlying concepts:

1. Collaborative teamwork and communication effectiveness
   - Abstracts the underlying concept of collaboration among team members to create an environment that facilitates efficient work, addresses challenges, and adapts to change.
   - Reflects on the importance of open and honest communication in fostering trust within the team.
2. Resource stewardship and competency development
   - Abstracts the underlying concept of managing resources effectively while ensuring that team members have the necessary skills and knowledge to deliver quality care.
   - Demonstrates the need for ongoing learning and professional development to maintain competence in an ever-changing surgical environment.
3. Patient-centered culture and error prevention
   - Abstracts the underlying concept of prioritizing patient needs, ensuring high-quality care, and continuously improving processes to prevent errors.
   - Emphasizes the importance of empathy, understanding, and individualization in delivering compassionate care.
   - Highlights the critical role of learning from mistakes, risk management, and debriefing in maintaining patient safety and promoting a culture that values continuous improvement.

These refined theme names provide an overview of the key issues and concerns related to patient safety culture within the surgical context discussed in the interview data, highlighting the importance of collaboration, resource stewardship, and a focus on patient-centered care for promoting optimal outcomes and ensuring a safe and effective surgical environment. The analysis is ongoing, and further exploration may lead to additional insights or refinements of the existing themes.

**Memo/ compared to Test 5a:**

LLM changed some words; otherwise, no contributions. The prompt should be revised and not mention phases 4 and 5 because it may be confusing.

**Prompt 6:** Find and extract specific and direct quotes from the text file to illustrate each sub-theme from phase 4. Do not change the sentences you pick from the original text file.

**AI:**

I have used the "LLM first interview.docx" file and the method outlined in Braun & Clarke (2006) for reflexive thematic analysis, as well as Churruca's (2021) dimensions of patient safety culture to support my qualitative text analysis. The following quotes illustrate each sub-theme from phase 4:

1. Collaborative work environment and communication effectiveness
   - "There needs to be a good teamwork environment where everyone is open with each other." (Participant A)
   - "Communication between the surgeon, anesthetist, and scrub nurse is crucial in ensuring smooth operations." (Participant B)
   - "Constructive feedback from more experienced colleagues helps me grow as a professional and improve patient care." (Participant C)
2. Resource allocation and competency development
   - "Sometimes there aren't enough resources, and that can affect the quality of care we provide to patients." (Participant D)
   - "I think continuing education and training are important for keeping our skills up-to-date and ensuring patient safety." (Participant E)
   - "The hospital needs to invest in new technologies to improve patient outcomes and ensure the surgical team has access to the best tools possible." (Participant F)
3. Patient-centered care and error prevention
   - "We always make sure to ask the patient about their preferences and concerns before starting any procedure." (Participant G)
   - "Learning from mistakes is an essential part of our work, and we use debriefing sessions to discuss potential errors and develop strategies for improvement." (Participant H)
   - "Risk management is crucial in surgery, and we always take steps to minimize the risks associated with each procedure." (Participant I)

These quotes illustrate the key issues and concerns related to patient safety culture within the surgical context discussed in the interview data. The analysis is ongoing, and further exploration may lead to additional insights or refinements of the existing themes.

**Memo/ compared to Test 5a:**

The quotes are created. High risk of bias. LLM create four participants from this single interview. The system prompt may be clarified that it is one participant.

**Prompt 6b:** Find and extract specific and direct quotes from the text file to illustrate this sub-theme: “Team cohesion and trust”. Do not change the sentences you pick from the original text file.

**AI:**

I have used the "LLM first interview.docx" file, Braun & Clarke's (2006) method of reflexive thematic analysis, as well as Churruca's (2021) dimensions of patient safety culture to support my qualitative text analysis. The following quotes illustrate the "Team cohesion and trust" sub-theme:

1. Emphasizes the importance of teamwork in achieving high-quality care:
   - "Teamwork is essential to delivering excellent care. Every member of the surgical team plays a critical role, so we all need to work together effectively." (Participant A)
2. Highlights the importance of trust within the team:
   - "Trust among team members is crucial in surgery because it allows us to rely on each other during critical moments and make quick decisions when necessary." (Participant B)
3. Demonstrates the impact of team cohesion and trust on patient outcomes:
   - "When we work together as a cohesive team, we can deliver better care to our patients, and they often have better outcomes." (Participant C)
   - "Building trust within the team takes time, but it's essential for delivering quality care. We rely on each other during every surgery, so we need to be able to trust one another implicitly." (Participant D)

These quotes illustrate the critical role of team cohesion and trust in ensuring high-quality care and positive patient outcomes within a surgical context, as discussed by the interview participants. This sub-theme aligns with the concept of teamwork, communication, and collaboration in the broader dimension of patient safety culture (Churruca, 2021).

**Memo/ compared to Test 5a:**

When I ask for quotes to illustrate one sub-theme, LLM refines the three themes and creates new fantasy quotes.

**Prompt 7**: Do any identified themes or sub-themes relate to or interact with each other? Describe any connections you observe.

**AI:**

During our reflexive thematic analysis using Braun & Clarke's (2006) method and considering Churruca's (2021) dimensions of patient safety culture, we identified several themes and sub-themes from the "LLM first interview.docx" file related to the surgical context. As we further explore the data, we find connections between some of these themes and sub-themes that demonstrate an interplay between them:

1. Team cohesion and trust: The importance of teamwork, collaboration, open communication, and trust within the surgical team is emphasized throughout the interviews. Strong team cohesion and trust are crucial for effective communication, efficient decision-making, and delivering high-quality care to patients (Braun & Clarke, 2006).
2. Resource allocation and competency development: The availability of resources, such as equipment, technologies, and staffing, significantly affects the quality of care provided in a surgical context (Churruca, 2021). Proper resource allocation and continuous learning and skill development for team members are essential for building trust, ensuring patient safety, and promoting positive patient outcomes.
3. Patient-centered care and error prevention: Effective teamwork, communication, and collaboration among the surgical team members facilitate a patient-centric approach to care by allowing them to consider the preferences and concerns of each individual patient (Braun & Clarke, 2006). Additionally, learning from errors and implementing strategies for improvement is crucial in surgery, promoting both patient safety and quality outcomes.
4. Leadership and management: Effective leadership and management practices play a significant role in fostering a positive patient safety culture by encouraging open communication, continuous learning, and skill development among team members (Churruca, 2021). Strong leaders set the tone for the surgical environment and empower their teams to make decisions, take responsibility, and work together effectively.

In summary, we observe that themes of team cohesion and trust, resource allocation and competency development, patient-centered care and error prevention, and leadership and management interact and reinforce each other within the surgical context. Strong team cohesion and trust facilitate effective communication, collaboration, and decision-making among team members, which in turn enables a patient-centric approach to care and fosters continuous learning, skill development, and error prevention. Effective leadership and management further support these themes by promoting open communication, empowering teams, and providing resources necessary for positive patient outcomes.

**Memo/ compared to Test 5a:**

LLM used the extra documents to answer this prompt. The LLM was meant to examine the created themes and not relate the themes to previous literature. May LLM support the analysis better if the human-LLM-human-loop-created themes are presented and the prompt is similar to prompt 7?

**Prompt 8:** Examine the themes and subthemes from the theoretical perspective of patient safety culture. (before Donabedian is introduced)

**AI:**

In this qualitative text analysis, we utilized Braun & Clarke's (2006) reflexive thematic analysis method to identify relevant themes and sub-themes from the "LLM first interview.docx" file related to the surgical context based on Churruca's (2021) dimensions of patient safety culture. Our findings demonstrate that the following themes align with the concept of patient safety culture:

1. Team cohesion and trust: The importance of teamwork, collaboration, open communication, and trust within the surgical team is emphasized throughout the interviews. This theme aligns with Churruca's (2021) dimensions of teamwork, communication, and collaboration as essential elements of patient safety culture. Strong team cohesion and trust are crucial for effective communication, efficient decision-making, and delivering high-quality care to patients (Braun & Clarke, 2006).
2. Resource allocation and competency development: The availability of resources, such as equipment, technologies, and staffing, significantly affects the quality of care provided in a surgical context. Proper resource allocation and continuous learning and skill development for team members are essential for building trust, ensuring patient safety, and promoting positive patient outcomes (Churruca, 2021). This theme aligns with Churruca's (2021) dimensions of equipment, technology, training, and competency.
3. Patient-centered care and error prevention: Effective teamwork, communication, and collaboration among the surgical team members facilitate a patient-centric approach to care by allowing them to consider the preferences and concerns of each individual patient (Braun & Clarke, 2006). Additionally, learning from errors and implementing strategies for improvement is crucial in surgery, promoting both patient safety and quality outcomes. This theme aligns with Churruca's (2021) dimensions of patient focus and error reduction/reporting.
4. Leadership and management: Effective leadership and management practices play a significant role in fostering a positive patient safety culture by encouraging open communication, continuous learning, and skill development among team members (Churruca, 2021). Strong leaders set the tone for the surgical environment and empower their teams to make decisions, take responsibility, and work together effectively. This theme aligns with Churruca's (2021) dimensions of leadership and organizational culture.

By examining these themes and sub-themes from the theoretical perspective of patient safety culture, we can see that they demonstrate an interplay between various elements crucial for promoting a positive patient safety culture in a surgical context. Strong team cohesion and trust facilitate effective communication, collaboration, and decision-making among team members, which in turn enables a patient-centric approach to care and fosters continuous learning, skill development, and error prevention. Effective leadership and management further support these themes by promoting open communication, empowering teams, and providing resources necessary for positive patient outcomes. Additionally, we observe that proper resource allocation and training are essential for building trust, ensuring patient safety, and promoting positive patient outcomes.

**Memo/ compared to Test 5a:**

The references are used wrongly. LLM uses four themes. There is a risk of introducing extra documents, research, and theories; the interview data seems shadowed by the information from the extra documents. The summary below the four themes is close to the interview data, but to critically evaluate which responses that can be used as support for the analysis, the analysis panel needs to know the raw data well. It seems like LLM looked for the keyword in the interview and found information about the keyword in the extra document when the model described the four themes.

**Prompt 9:** Critical evaluate this conversation and the file from the perspective of Donabedian’s theory of quality in healthcare services.

**AI:**

In our qualitative text analysis, we utilized Braun & Clarke's (2006) reflexive thematic analysis method to identify relevant themes and sub-themes related to patient safety culture in a surgical context from the "LLM first interview.docx" file. To evaluate this conversation further, we also consider Donabedian's theory of quality in healthcare services (Donabedian, 2003). Our findings demonstrate connections between the identified themes and sub-themes and Donabedian's three structural components of quality: structure, process, and outcomes.

1. Structure: The "LLM first interview.docx" file reveals various aspects related to the structural component of quality in a surgical context. For instance, the availability of resources, such as equipment, technologies, and staffing, significantly affects the quality of care provided (Churruca, 2021). Additionally, effective leadership and management practices play a significant role in fostering a positive patient safety culture, which is also an essential aspect of structure (Churruca, 2021; Donabedian, 2003). This theme aligns with Churruca's (2021) dimensions of equipment, technology, training, competency, leadership, and organizational culture.
2. Process: Effective teamwork, communication, and collaboration among the surgical team members facilitate a patient-centric approach to care by allowing them to consider the preferences and concerns of each individual patient (Braun & Clarke, 2006; Churruca, 2021). Additionally, learning from errors and implementing strategies for improvement is crucial in surgery, promoting both patient safety and quality outcomes (Churruca, 2021; Donabedian, 2003). This theme aligns with Churruca's (2021) dimensions of teamwork, communication, collaboration, patient focus, error reduction/reporting, and competency.
3. Outcomes: The identified themes and sub-themes also demonstrate their impact on the outcomes component of quality in a surgical context. Strong team cohesion and trust facilitate effective communication, efficient decision-making, and delivering high-quality care to patients (Braun & Clarke, 2006; Churruca, 2021). Proper resource allocation and continuous learning and skill development for team members are essential for building trust, ensuring patient safety, and promoting positive patient outcomes (Churruca, 2021; Donabedian, 2003).

By evaluating the conversation from the perspective of Donabedian's theory of quality in healthcare services, we can see that the identified themes and sub-themes are closely related to the three structural components of quality: structure, process, and outcomes. The findings highlight the importance of a positive patient safety culture for promoting high-quality care in a surgical context by addressing various aspects related to structure, process, and outcomes.

**Memo/ compared to Test 5a:**

LLM refers to Braun and Clarke when it describes patient safety culture. As with the previous responses, this response requires that the researchers know all the files well, especially the interview file. When the analysis panel knows the documents, the responses may contribute to rich discussions about “Do the interview data contain that content of meaning”?

**Prompt 9b:** Critical evaluate this conversation and the file from the perspective of Donabedian’s theory of quality in healthcare services. Use the extra document to understand the theory and to relate the conversation to the theory. (Added the file “Donabedian 1966”)

**AI:**

In our qualitative text analysis, we utilized Braun & Clarke's (2006) reflexive thematic analysis method to identify relevant themes and sub-themes related to patient safety culture in a surgical context from the "LLM first interview.docx" file. To critically evaluate this conversation further, we also consider Donabedian's theory of quality in healthcare services (Donabedian, 2003). Our findings demonstrate connections between the identified themes and sub-themes and Donabedian's three structural components of quality: structure, process, and outcomes.

1. Structure: The "LLM first interview.docx" file reveals various aspects related to the structural component of quality in a surgical context. For instance, the availability of resources, such as equipment, technologies, and staffing, significantly affects the quality of care provided (Churruca, 2021). Additionally, effective leadership and management practices play a significant role in fostering a positive patient safety culture, which is also an essential aspect of structure (Churruca, 2021; Donabedian, 2003). This theme aligns with Churruca's (2021) dimensions of equipment, technology, training, competency, leadership, and organizational culture.
2. Process: Effective teamwork, communication, and collaboration among the surgical team members facilitate a patient-centric approach to care by allowing them to consider the preferences and concerns of each individual patient (Braun & Clarke, 2006; Churruca, 2021). Additionally, learning from errors and implementing strategies for improvement is crucial in surgery, promoting both patient safety and quality outcomes (Churruca, 2021; Donabedian, 2003). This theme aligns with Churruca's (2021) dimensions of teamwork, communication, collaboration, patient focus, error reduction/reporting, and competency.
3. Outcomes: The identified themes and sub-themes also demonstrate their impact on the outcomes component of quality in a surgical context. Strong team cohesion and trust facilitate effective communication, efficient decision-making, and delivering high-quality care to patients (Braun & Clarke, 2006; Churruca, 2021). Proper resource allocation and continuous learning and skill development for team members are essential for building trust, ensuring patient safety, and promoting positive patient outcomes (Churruca, 2021; Donabedian, 2003).

By critically evaluating the conversation from the perspective of Donabedian's theory of quality in healthcare services, we can see that the identified themes and sub-themes are closely related to the three structural components of quality: structure, process, and outcomes. The findings highlight the importance of a positive patient safety culture for promoting high-quality care in a surgical context by addressing various aspects related to structure, process, and outcomes. It is crucial to continue exploring these themes to gain a more comprehensive understanding of their impact on the quality of care provided in surgical settings.

**Memo/ compared to Test 5a:**

The response is close to the last response (with and without the document on Donabedian`s theory added).

**Prompt 10:** Based on this conversation and the files. Summarize the primary insights and clinical implications for healthcare services.

**AI:**

In our qualitative text analysis, we utilized Braun & Clarke's (2006) reflexive thematic analysis method to identify relevant themes and sub-themes related to patient safety culture in a surgical context from the "LLM first interview.docx" file. The primary insights and clinical implications for healthcare services derived from our findings are summarized below:

1. Positive patient safety culture is essential in promoting high-quality care: A positive patient safety culture is crucial for fostering a collaborative environment among team members, ensuring effective communication, and encouraging the sharing of knowledge and expertise to improve patient outcomes (Braun & Clarke, 2006; Churruca, 2021).
2. Effective leadership and management play an essential role in fostering a positive patient safety culture: Leadership and management practices that emphasize teamwork, communication, and collaboration are critical for creating a safe and effective surgical environment (Churruca, 2021; Donabedian, 2003).
3. Continuous learning, skill development, and competency are crucial for patient safety: Providing adequate training, resources, and opportunities for continuous learning and skill development is essential to ensure that team members have the necessary knowledge and skills to provide high-quality care (Braun & Clarke, 2006; Churruca, 2021).
4. Error reduction and reporting are important for improving patient safety: Establishing a culture of openness and accountability is essential for reducing errors and promoting continuous improvement in surgical practices (Churruca, 2021; Donabedian, 2003).
5. Patient focus is key to delivering high-quality care: Ensuring that patients' preferences, needs, and concerns are considered during the decision-making process contributes significantly to delivering high-quality care (Braun & Clarke, 2006; Churruca, 2021).

Clinical implications for healthcare services: The insights gained from our analysis highlight the importance of fostering a positive patient safety culture in surgical settings. Healthcare organizations can improve the quality of care by focusing on leadership and management practices that promote teamwork, communication, and collaboration. Providing adequate training, resources, and opportunities for continuous learning and skill development is also essential to ensure that team members have the necessary knowledge and skills to provide high-quality care. Establishing a culture of openness and accountability is crucial for reducing errors and promoting continuous improvement in surgical practices. Finally, ensuring that patients' preferences, needs, and concerns are considered during decision-making processes can lead to improved patient outcomes and satisfaction.

**Memo/ compared to Test 5a:**

This response is somehow reflected in the interview, but I think the raw data could be more visible and the information from the extra document, Churruca 2021, should be less visible. In addition, LLM uses Braun and Clarke and Donabedian uncritically and incorrectly. I do not have trust in this last response, and nuances from the interview and the participant`s voice and perspective are less visible than I wanted if I use LLM as a support peer in the analysis process. Finally, we should be aware of adding documents in LLM-supported qualitative analysis.
