# Supplementary material for: Reflecting on LLM Support in Reflexive Thematic Analysis: An Exploratory Study
Source: Qual Health Res. 2025 Sep 8;36(2-3):191–205. doi: 10.1177/10497323251365211 (PMC12949038; doi:10.1177/10497323251365211)
Supplement: Supplemental Material - Reflecting on LLM Support in Reflexive Thematic Analysis: An Explorative Study [file sj-zip-1-qhr-10.1177_10497323251365211.zip › Supplemental_Material_Items.docx]

# Supplemental Material Items

Your template for structuring the title and description of supplemental files is fine with me:

| **Title** | **Description** |
| --- | --- |
| [Supplemental_File_1_Test_1_Norwegian] - Supplemental material for [Reflecting on LLM-Support in Reflexive Thematic Analysis: An Explorative Study] | Supplemental material, [Supplemental_File_1_Test_1_Norwegian], for [Reflecting on LLM-Support in Reflexive Thematic Analysis: An Explorative Study] by [Authors’ names] in [SAGE Journals’ Qualitative Health Research] |
| [Supplemental_File_2_Test_1_English] - Supplemental material for [Reflecting on LLM-Support in Reflexive Thematic Analysis: An Explorative Study] | Supplemental material, [Supplemental_File_2_Test_1_English], for [Reflecting on LLM-Support in Reflexive Thematic Analysis: An Explorative Study] by [Authors’ names] in [SAGE Journals’ Qualitative Health Research] |
| [Supplemental_File_3_Test_2_Norwegian] - Supplemental material for [Reflecting on LLM-Support in Reflexive Thematic Analysis: An Explorative Study] | Supplemental material, [Supplemental_File_3_Test_2_Norwegian], for [Reflecting on LLM-Support in Reflexive Thematic Analysis: An Explorative Study] by [Authors’ names] in [SAGE Journals’ Qualitative Health Research] |
| [Supplemental_File_4_Test_2_English] - Supplemental material for [Reflecting on LLM-Support in Reflexive Thematic Analysis: An Explorative Study] | Supplemental material, [Supplemental_File_4_Test_2_English], for [Reflecting on LLM-Support in Reflexive Thematic Analysis: An Explorative Study] by [Authors’ names] in [SAGE Journals’ Qualitative Health Research] |
| [Supplemental_File_5_Test_3] - Supplemental material for [Reflecting on LLM-Support in Reflexive Thematic Analysis: An Explorative Study] | Supplemental material, [Supplemental_File_5_Test_3], for [Reflecting on LLM-Support in Reflexive Thematic Analysis: An Explorative Study] by [Authors’ names] in [SAGE Journals’ Qualitative Health Research] |
| [Supplemental_File_6_Test_4] - Supplemental material for [Reflecting on LLM-Support in Reflexive Thematic Analysis: An Explorative Study] | Supplemental material, [Supplemental_File_6_Test_4], for [Reflecting on LLM-Support in Reflexive Thematic Analysis: An Explorative Study] by [Authors’ names] in [SAGE Journals’ Qualitative Health Research] |
| [Supplemental_File_7_Test_5a] - Supplemental material for [Reflecting on LLM-Support in Reflexive Thematic Analysis: An Explorative Study] | Supplemental material, [Supplemental_File_7_Test_5a], for [Reflecting on LLM-Support in Reflexive Thematic Analysis: An Explorative Study] by [Authors’ names] in [SAGE Journals’ Qualitative Health Research] |
| [Supplemental_File_8_Test_5b] - Supplemental material for [Reflecting on LLM-Support in Reflexive Thematic Analysis: An Explorative Study] | Supplemental material, [Supplemental_File_8_Test_5b], for [Reflecting on LLM-Support in Reflexive Thematic Analysis: An Explorative Study] by [Authors’ names] in [SAGE Journals’ Qualitative Health Research] |
| [Supplemental_File_9_Test_5c] - Supplemental material for [Reflecting on LLM-Support in Reflexive Thematic Analysis: An Explorative Study] | Supplemental material, [Supplemental_File_9_Test_5c], for [Reflecting on LLM-Support in Reflexive Thematic Analysis: An Explorative Study] by [Authors’ names] in [SAGE Journals’ Qualitative Health Research] |
| [Supplemental_File_10_Test_5d] - Supplemental material for [Reflecting on LLM-Support in Reflexive Thematic Analysis: An Explorative Study] | Supplemental material, [Supplemental_File_10_Test_5d], for [Reflecting on LLM-Support in Reflexive Thematic Analysis: An Explorative Study] by [Authors’ names] in [SAGE Journals’ Qualitative Health Research] |
| [Supplemental_File_11_Test_6] - Supplemental material for [Reflecting on LLM-Support in Reflexive Thematic Analysis: An Explorative Study] | Supplemental material, [Supplemental_File_11_Test_6], for [Reflecting on LLM-Support in Reflexive Thematic Analysis: An Explorative Study] by [Authors’ names] in [SAGE Journals’ Qualitative Health Research] |
| [Supplemental_File_12_Test_7] - Supplemental material for [Reflecting on LLM-Support in Reflexive Thematic Analysis: An Explorative Study] | Supplemental material, [Supplemental_File_12_Test_7], for [Reflecting on LLM-Support in Reflexive Thematic Analysis: An Explorative Study] by [Authors’ names] in [SAGE Journals’ Qualitative Health Research] |
| [Supplemental_File_13_Consent_Form] - Supplemental material for [Reflecting on LLM-Support in Reflexive Thematic Analysis: An Explorative Study] | Supplemental material, [Supplemental_File_13_Consent_Form], for [Reflecting on LLM-Support in Reflexive Thematic Analysis: An Explorative Study] by [Authors’ names] in [SAGE Journals’ Qualitative Health Research] |
